# HIVIL: A human *in vitro* inflammatory liver model recapitulates immune-associated drug effects with high predictivity

**DOI:** 10.1016/j.namjnl.2025.100032

**Published:** 2025-06-24

**Authors:** Xiaozhong Huang, Yun Ting Soong, Jiahao Wang, Claire Jia Yi Ng, Kartik Mitra, Farah Tasnim, Hanry Yu

**Affiliations:** a Institute of Bioengineering and Bioimaging, #07-01, 31 Biopolis Way, The Nanos, Singapore 138669, Singapore; b Biomedical Sciences Industry Partnership Office (BMSIPO), A∗STAR, 4 Fusionopolis Way, Kinesis #09-11, Singapore 138635, Singapore; c Department of Physiology, The Institute of Digital Medicine (WisDM), Yong Loo Lin School of Medicine, National University of Singapore, MD9-04-11, 2 Medical Drive, Singapore 117593, Singapore; d CAMP, Singapore-MIT Alliance for Research and Technology, 1 CREATE Way, Level 4 Enterprise Wing, Singapore 138602, Singapore; e Mechanobiology Institute, National University of Singapore, T-Lab, #05-01, 5A Engineering Drive 1, Singapore 117411, Singapore

**Keywords:** Drug-induced liver injury, hiPSC-derived Kupffer cells, *In vitro* liver model, Cytokines

## Abstract

DILI (Drug Induced Liver Injury) is one of leading cause of failure in drug development due to adverse reaction outcomes and health hazards. Besides, understanding DILI is challenging due to lack of relevant *in vitro* models] that recapitulate human *in vivo* physiological responses. Current *in vitro* models employing primary human Kupffer cells (PHKCs) or alternative cells such as THP-1 derived macrophages are either complex or do not recapitulate physiological drug-induced cytokine responses. We leveraged on human iPSC derived Kupffer cells (iKCs) that functionally resemble PHKCs to establish a human *in vitro* inflammatory liver model (HIVIL) that is complex enough to be physiological and simple enough to be robust. HIVIL, comprising of iKCs co-cultured with iPSCs derived hepatocytes can recapitulate physiological levels of DILI associated inflammatory response of known DILI drugs *in vitro*. Out of 18 drug candidates tested, the cytokine responses of 16 drugs correlated (88.9 %) well with the reported serum cytokine profiles of DILI patients implying a closer-to-physiological relevant immune responses and cytochrome P450 expression . Moreover, HIVIL model was able to mechanistically distinguish the TNF⍺ mediated hepatotoxic effect of Trovafloxacin over Levofloxacin. RNA-Seq analysis provided further insight into the interactions between the cytokines and drug-induced liver injury. In contrast, HIVIL using THP-1 derived macrophages instead of iKCs did not recapitulate the cytokine responses upon treatment with paradigm compounds, demonstrating the importance of KCs-produced cytokines on hepatocyte xenobiotic metabolism. In summary, our study demonstrates for the first time, the use of iKCs and iHeps as a simple, robust and physiologically relevant *in vitro* drug testing model for DILI candidates.

## 1 Introduction

Drug-induced liver injury (DILI) is a leading cause of drug attrition in development, post-marketing drug withdrawal, and black box warnings by regulatory agencies. Besides, DILI also poses serious threat to human health by altering the hepatic immune landscape predisposing to inflammation and immune mediated toxicity (Waddington et al., 2018). DILI is broadly classified into intrinsic DILI and idiosyncratic DILI (iDILI). The former is predictable and exhibits a dose-dependent hepatotoxic effect, whereas the latter exhibits a dose-independent hepatotoxic effect arising due to altered hepatic immune landscape. iDILI is further classified into four types based on clinical representation, namely, type-1: drug-induced autoimmune hepatitis, type-2: HLA-associated DILI, type-3: anti-cytochrome P450 autoimmune DILI and type-4: immune DILI associated with systemic immune reactions like Stevens–Johnson Syndrome (SJS) and Toxic Epidermal Necrolysis (TEN) (Teschke, 2025). Despite their differences in mechanisms and clinical representation, some suggests intrinsic DILI and iDILI are more alike on the pre-text of stress and dose-response principles (Ganey et al., 2004a; Roth and Ganey, 2010). Stress or inflammatory insult (Ex. LPS exposure) can sensitize the liver microenvironment predisposing to liver injury, which can make otherwise tolerable/therapeutic drug dosage to elicit iDILI reactions (Shaw et al., 2007). Currently, DILI is clinically evaluated *in vivo* based on Roussel Uclaf Causality Assessment Method (RUCAM) (Danan and Teschke, 2019). However, pre-clinical identification of iDILI candidates remains challenging. Majority of intrinsic DILI candidates are eliminated at pre-clinical testing stages, on the contrary pre-clinical identification of potential iDILI candidates remains challenging due to its complex mechanism and lack of reliable models (Kaplowitz, 2005; Zhou et al., 2019).

Liver is an multi-modal metabolic and immunological organ, harboring a vast array of innate and adaptive immune cells (Waddington et al., 2018). Liver resident macrophages or Kupffer cells (KCs), are the primary immune responders mediating inflammatory responses. KCs respond to hepatic injury by secreting a substantial number of cytokines such as TNF⍺, IL-6, IL1β..etc that regulate DILI propagation, metabolic activity of hepatocytes and hence, in turn, alter hepatic immune microenvironment (Shan and Ju, 2019). Consequentially, measurement of such cytokines in patients has shown predictive value for prognosis of DILI in patients (Steuerwald et al., 2013). Animal models have also substantially contributed to mechanistic understanding of DILI. However, costs, ethical concerns and growing demands on reducing, refining and replacing use of animals have highlighted the need for relevant alternative *in vitro* models (Tasnim et al., 2021). In addition, discrepancies in DILI observation emerge frequently in rodent based models due to species and physiological difference. For instance, some studies show Trovofloxacin (TVX)-induced TNFα inhibition mice models which is in contrary to TVX-induced TNFα increase observed in humans *in vivo* (Bonzo et al., 2015; Rose et al., 2016; Shaw et al., 2010)). Alternatively, primary human KCs (PHKCs) are utilized to develop relevant models that recapitulate human physiological response. However, the major bottleneck of availability, costs, ethical concerns and batch-batch variability remains a major concern (Kegel et al., 2015). For instance, commercially established models such as 3D InSight™ Human Liver Microtissue (InSphero) and HepatoMune™ (Hepregen/BioIVT) rely on PHKCs and lack high-throughput capacity. Attempts to replace PHKCs with THP-1 or peripheral monocyte-derived macrophages, has failed due to their differences in macrophage ontogeny, function, cytokine production profile and lack of liver-specific imprinting (Kermanizadeh et al., 2019; Bonnardel et al., 2019; Mestas and Hughes, 2004). For instance, co-culture deploying THP-1derived macrophages showed a decrease in TNFα, when treated with Troglitazone, which is contrary to observed clinical mechanism of TNF⍺-induced apoptosis by Troglitazone (Edling et al., 2009). In this study, we leverage on our previously published protocol for generating mature Kupffer cells from human induced pluripotent stem cells (iKCs). The iKCs were co-cultured with hepatocytes and optimized to develop a human *in vitro* inflammatory liver model (HIVIL) that can recapitulate DILI response *in vitro*. The HIVIL model was validated with 18 known DILI drugs, demonstrating its capability to replicate the drug-induced immune response accurately with physiologically relevant cytokine profiles.

## 2 Results

### 2.1 Establishing and characterization of HIVIL: human *in vitro* inflammatory liver model

First we optimized our previously described method of generating induced pluripotent stem cell-derived Kupffer cells (iKCs) (Tasnim et al., 2019) through systematic characterization of serum and growth factor supplementation to improve cell yield. Addition of macrophage colony-stimulating factor (M-CSF) together with serum resulted in the highest cell density/yield and enhanced expression of macrophage and KC-specific markers (Fig. S1). Serum was essential for initial cell attachment, and the combination of serum with M-CSF improved cell yield compared to serum alone without compromising cell function. Thus, we selected serum plus M-CSF conditions for subsequent experiments.

**Fig. 1:**
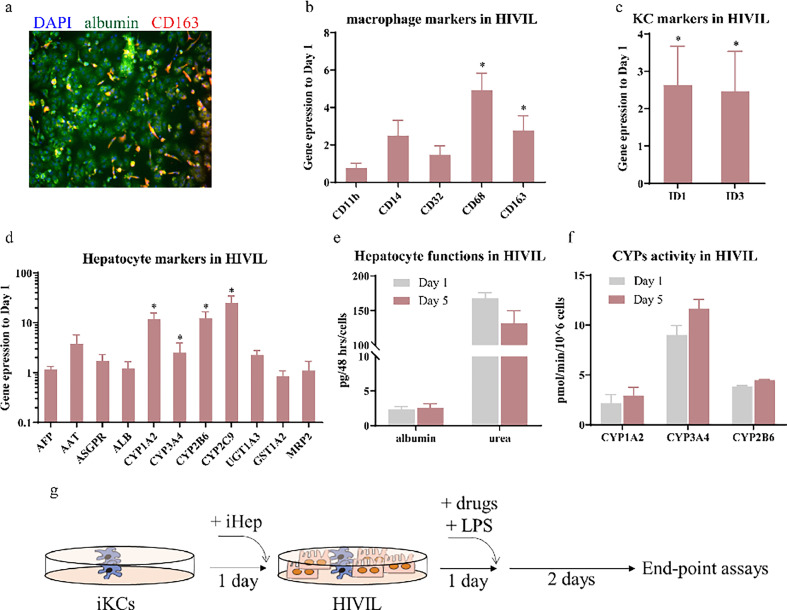
Functional characterization of co-culture and development of HIVIL (a) Co-existence of iPSC-derived hepatocyte (iHeps, albumin, green) and Kupffer cells (iKCs, CD163, red) in HIVIL on Day 5 of co-culture. Gene expression profile of (b) macrophage markers, (c) KC-specific markers and (d) hepatocyte markers on Day 5 as measured by qPCR. Relative expression of tested genes on Day 5 was normalized to Day 1. (e) Albumin and urea, as well as (f) CYP activity were measured on Day 1 and Day 5. Functional activity was normalized to cell numbers. *: *p <* 0.05, **: *p <* 0.01; asterisks in b, c and d represent comparison to gene expression on Day 1. (g) Schematic of HIVIL for drug testing. Error bars represent s.e.m, *n* = 3.

We next tested if iKCs generated using our refined protocol could be effectively co-cultured with hiPSC-derived hepatocytes (iHeps) while maintaining both cell types' functionalities. Co-staining with CD163 and albumin on Day 5 confirmed successful maintenance of both cell populations throughout the experimental period (Fig. 1a). Marker and functional characterization showed maintenance or improvement in macrophage markers by 1.5- to 5-fold (CD11b: 0.8±0.2-fold, Fig. 1b). Importantly, KC-specific markers ID1 and ID3, distinguishing KCs from non-liver macrophages, were upregulated by 2.6- and 2.4-fold, respectively (Fig. 1c). Similarly, hepatocyte markers were maintained (GST1A2: 0.8±0.2-fold; MRP2/AFP: 1.1-fold) or improved by 1.7- to 24-fold (Fig. 1d). Functional assays measuring metabolic activity of iHeps, including albumin and urea production, showed no significant changes (albumin: 2.4–2.6 pg/cell/48 hr; urea: 131–168 pg/cell/48 hr) (Fig. 1e). Cytochrome P450 (CYP) activities, CYP1A2 (2.7 vs. 2.3 pmol/million cells/min), CYP3A4 (8.3 vs. 11 pmol/million cells/min), and CYP2B6 (3.9 vs. 4.5 pmol/million cells/min), remained stable during co-culture (Fig. 1f). To integrate the co-culture into the HIVIL model, we carefully optimized inflammation stimulation conditions, comparing two reported stimulators: interleukin-2 (IL-2) (Bosco et al., 2000) and lipopolysaccharide (LPS) (Bonzo et al., 2015; Nguyen et al., 2015; Rose et al., 2016). IL-2 failed to stimulate cytokine production in primary human KCs and iKCs (Fig. S2a); hence, LPS was selected for subsequent inflammatory stimulation experiments, aligning with previous findings (Nguyen et al., 2015). Given significant variability in reported LPS stimulation durations, we evaluated time-dependent inflammatory responses, identifying 48 h as optimal (Fig. S2b). The finalized experimental design is presented in Fig. 1g.

**Fig. 2:**
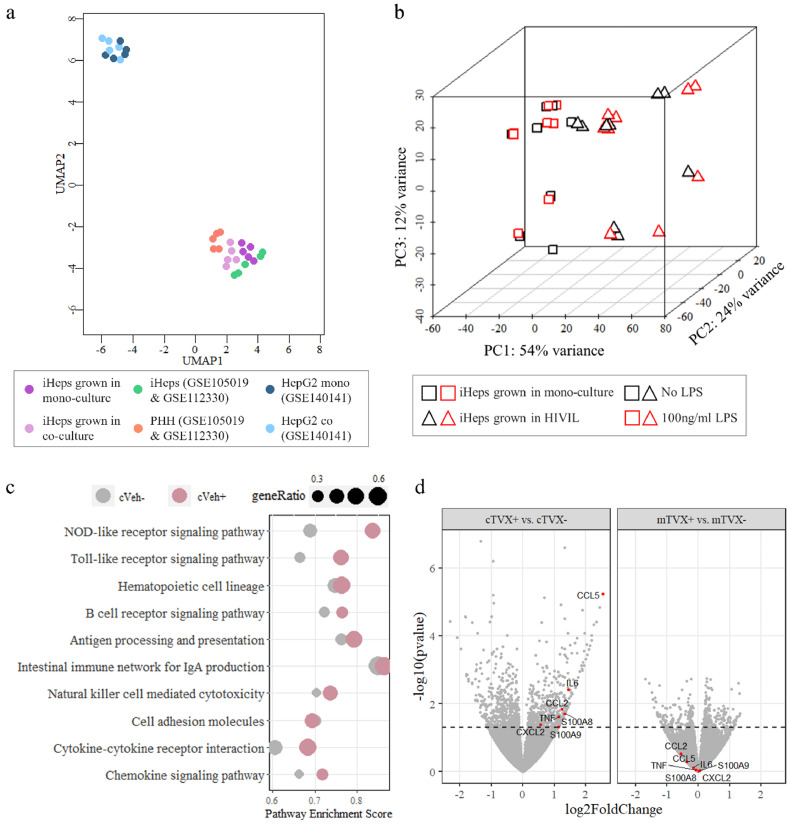
Effect of different model variables in HIVIL. (a) UMAP-based dimensionality reduction plot comparing iHeps sequenced in this study against hepatocytes from other studies. (b) PCA plot demonstrating two model variables: iHeps grown in mono-culture vs. in HIVIL (square vs. triangle), LPS presence vs. absence (black vs. red outline). (c) Top 10 KEGG pathways enriched in iHeps co-cultured with iKC (cVeh- & cVeh+ vs. mVeh-). Gene ratio refers to the number of genes in the leading edge driving the pathway enrichment, divided by the total number of genes in the pathway and are expressed in the sample. Samples were named as: <mono-or co-culture (m or c)> <treatment (Veh or TVX or LVX)> <absent or including LPS (- or +)>. (d) Volcano plots to compare the impact of LPS across different conditions. The dotted line represents p-value = 0.05.

To understand the effects of model variables in HIVIL, transcriptomic analyses via RNA-Seq were conducted. After 48 h of drug or vehicle treatment, CD163-negative iHeps were isolated from mono-cultures and HIVIL for sequencing via magnetic bead sorting. Transcriptomes of our vehicle-treated iHeps were compared to commonly used hepatocyte cell line HepG2 (Padberg et al., 2020), PHHs and iHeps from other studies (Fu et al., 2019; Xie et al., 2019) (Fig. 2a). The Uniform Manifold Approximation and Projection (UMAP) plot showed our iHeps (mono-culture and HIVIL) clustered closely with PHH and state-of-the-art iHeps, distinct from HepG2 (Fig. 2a). Principal component analysis (PCA) indicated samples separated primarily by co-culture status rather than LPS treatment (Fig. 2b). Co-culture with iKCs significantly enriched immune-related pathways such as toll-like receptor and chemokine signaling in iHeps compared to mono-culture (Table S1; Fig. 2c). LPS-treated co-cultures (cVeh+) showed greater pathway enrichment than untreated co-cultures (cVeh-). LPS alone did not cause differential gene expression in mono-cultures, confirming its insufficient inflammatory impact without iKCs. Finally, we assessed if additional stressors, like immune-mediated drugs, could enhance inflammatory responses in HIVIL. Trovafloxacin (TVX), a known immune-mediated hepatotoxicant, significantly increased pro-inflammatory cytokine and chemokine mRNA expression in LPS-treated co-cultures but not mono-cultures (Fig. 2d). Overall, RNA-Seq analyses demonstrated that: (1) our iHeps were transcriptionally comparable to PHHs and high-quality iHeps; (2) co-culture significantly altered global gene expression compared to mono-cultures; (3) selected LPS conditions appropriately induced inflammatory responses without overstimulation; (4) key immune pathways were upregulated specifically in co-cultures, enhanced by LPS; and (5) immune-mediated drug exposure further amplified inflammatory effects. These results validate the optimized HIVIL model for future drug-testing applications.

### 2.2 HIVIL recapitulates clinically relevant cytokine responses of drug induced immune response

The HIVIL model was subjected to treatment with drugs known to cause immune/inflammation mediated liver damage and the *in vitro* cytokine responses (namely, IL-6, IL-10 and TNFα) of HIVIL were corroborated with the known clinical or *in vivo* responses (Table S2). The drug concentrations were chosen based on the Cmax of the compounds (upper limit capped to 20-fold Cmax). Drugs, namely amodiaquine (AQ), isoniazid (INH), lamotrigine (LTG), phenytoin (PHT), and diclofenac (DIC), were selected as training set. Additionally, TVX was also included in the training set because of its common use in DILI studies and association with immune responses (Bonzo et al., 2015; Rose et al., 2016; Shaw et al., 2010).

**Fig. 3:**
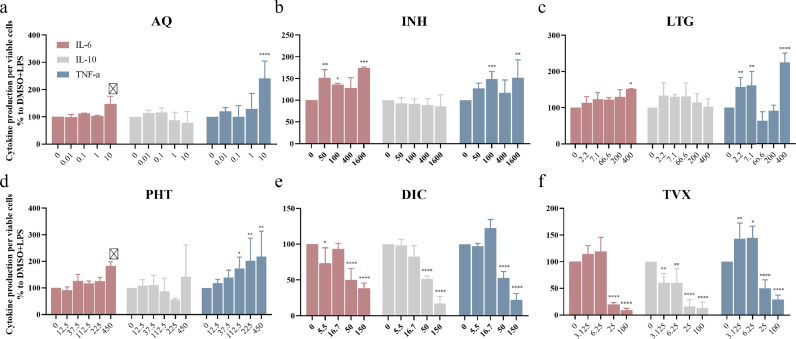
Changes in cytokines level upon treatment of 6 paradigm compounds used as training set. The level of interleukin-6 (IL-6), interleukin-10 (IL-10) and tumor necrosis factor α (TNFα) were examined in HIVIL and normalized to the corresponding cell viability. Data is expressed as the percentage of the LPS-treated vehicle control. Error bars represent s.e.m, n = 3. One-way ANOVA was applied. *p < 0.05, **p < 0.01, ***p < 0.001 and ****p < 0.0001 between treatment and vehicle control. ns: non-significant.

AQ, INH, LTG and PHT have been reported to cause an increase in IL-6 in patients with DILI (Steuerwald et al., 2013). These clinical findings were recapitulated in HIVIL, as demonstrated by the drug-induced IL-6 increase (Fig. 3a–d). AQ significantly induced IL-6 by 1.47-fold at 10 μM, while INH induced IL-6 by at least 1.2-fold at all doses tested. Both LTG) and PHT elicited a dose-dependent increase in IL-6 levels, ranging approximately 1.5- to 2-fold over baseline. Whereas in contrast, IL-10 responses were minimally affected by these drugs. Nonsteroidal anti-inflammatory drugs are frequent causes of immune-mediated drug responses and are represented by DIC in this study. DIC has been reported to mechanistically inhibit IL-6 production in humans (Gan, 2010). The clinical dose-dependent decrease of IL-6 was recapitulated in HIVIL, although a decrease of IL-10 was observed (Fig. 3e). TVX hepatotoxicity is associated with increased TNFα production (Shaw et al., 2010) and IL-6 production (Giustarini et al., 2019). In HIVIL, low dose of TVX (i.e., 3.125 μM and 6.25 μM) induced TNFα and IL-6 by 1.5-fold, although high doses (>25 μM) showed inhibitory effects on cytokine production (Fig. 3f). In addition, dose-dependent IL-10 decrease was observed upon TVX treatment.

Taken together, our results showed that HIVIL could recapitulate the reported human clinical IL-6 responses of 6 paradigm compounds, and TVX-specific TNFα induction, suggesting that HIVIL could provide additional insights into changes in cytokines that have not been reported *in vivo*.

### 2.3 Stratification of DILI compounds based on cytokine profile

**Fig. 4:**
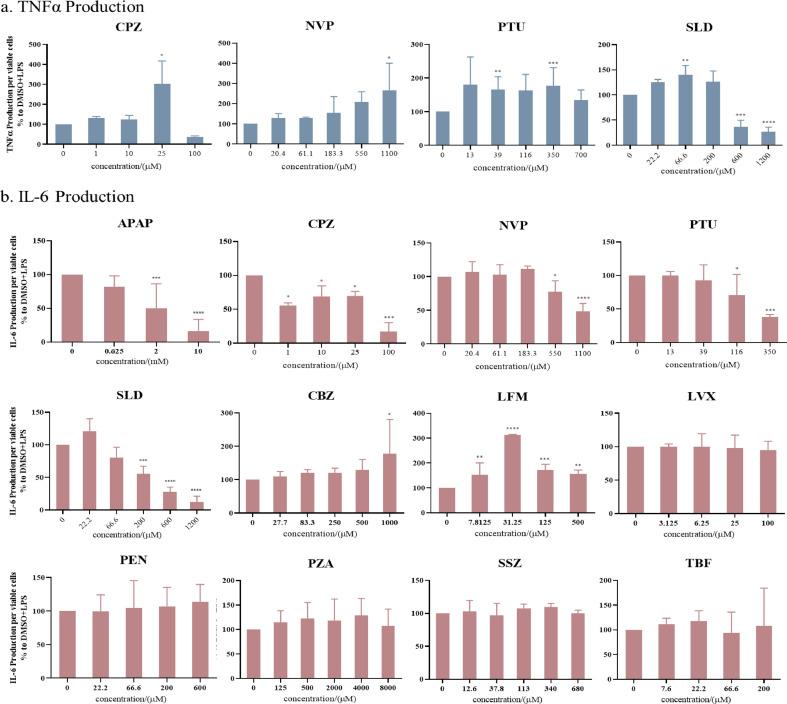
Changes in TNFα and IL-6 level upon treatment of 12 test compounds. The level of TNFα (blue bar, a) and IL-6 (red bar, b) was assessed in HIVIL treated with the drug stated in the plot title. Cytokine production was normalized to cell viability. Data is expressed as the percentage of the LPS-treated vehicle control. Error bars represent s.e.m, *n* = 3. One-way ANOVA was applied. *p < 0.05, **p < 0.01, ***p < 0.001 and ****p < 0.0001 between treatment and vehicle control. ns: non-significant.

We further expanded to test the cytokine effects of more reported hepatotoxic compounds that have been shown immune-mediated drug effects in animals (Table S2) to ascertain if the HIVL model can be employed to stratify the DILI compounds. TNFα and IL-6 production was considered as criteria to ascertain the compound response. Chlorpromazine (CPZ), nevirapine (NVP), propylthiouracil (PTU) and sulindac (SLD) are known to cause TNFα induction in animals (Table S2). Treatment with these drugs induced TNFα production in HIVIL (Fig. 4a). TNFα induction was observed in lower doses of CPZ (25 µM) or SLD (<200 µM), while it occurred in all concentrations of NVP or PTU tested. Based on drug-dependent IL-6 production, the tested compounds could be classified into three groups: (1) dose-dependent inhibition: APAP, CPZ, PTU, NVP and SLD; (2) dose-dependent induction: carbamazepine (CBZ) and leflunomide (LFM); and (3) no dose-dependent changes: levofloxacin (LVX), penicillin (PEN), pyrazinamide (PZA), sulfasalazine (SSZ) and terbinafine (TBF) (Fig. 4b). Among these changes, immune-mediated effects of APAP (Ganey et al., 2004b; Honarmand et al., 2012), SLD (Kang et al., 2001; Zou et al., 2009) and CBZ (Higuchi et al., 2012)), as well as the non-immune-mediated drugs LVX, PEN, PZA (Ho et al., 2015) and TBF (Ho et al., 2015) have been reported either clinically or in animal studies (Table S2) and successfully recapitulated in our *in vitro* model (including 50 % reduction of IL-6 upon treatment with APAP and SLD, 1.7-fold increase in IL-6 upon treatment with CBZ). In the contrast, LFM and SSZ, both of which have been reported as IL-6 suppressors, caused a 3.1-fold increase in LFM and no change in SSZ and hence were not correctly recapitulated in HIVIL. Overall, HIVIL correctly reproduced cytokine changes of 10 out of 12 drugs (Fig. 4). Combined with 6 training compounds (Fig. 3), HIVIL could correctly differentiate the potential drug’s effect on IL-6 and TNFα production in 16 out of the 18 compounds tested (88.9 %).

In order to ensure that the changes in cytokine levels were not due to changes in cell viability upon drug treatment, or variations in cytotoxicity to iKCs and iHeps, we normalized cytokine production to cell viability (Fig. 3, Fig. 4) and also tested the viability and cytokine production of iHep mono-culture and iKC mono-culture separately and compared it to the co-culture (HIVIL) (Fig. S3 a–d). The viability profiles were similar among iHeps, iKCs and HIVIL when treated with APAP, TVX or LVX, suggesting that any decrease in cytokine production upon treatment with these drugs cannot be attributed to overt toxicity to iKCs. Despite differences in viability between iKCs and HIVIL upon SLD and CPZ treatment, expected cytokine responses were observed only in HIVIL and not in iKCs alone (details in supplementary information). This suggests that changes in cytokine levels in HIVIL are not caused by effects of the drugs on iKCs alone but are also affected by the differential effects of parent compounds and processed products metabolized by hepatocytes. Hence, both cell types (iKCs for their cytokine-producing ability and iHeps for their drug metabolism activity) are critical for mimicking immune-mediated effects of drugs in HIVIL.

### 2.4 Decrease of IL-6 occurs at a close-to-physiological drug concentration compared to cell viability

**Fig. 5:**
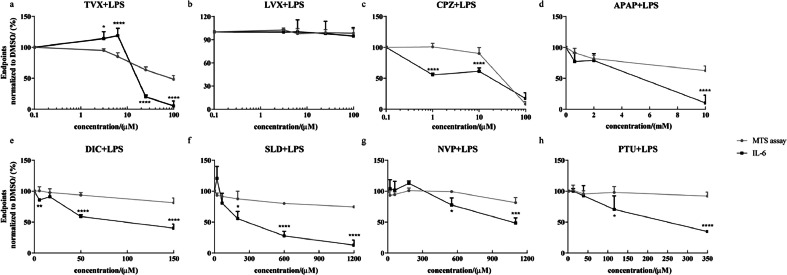
The dynamics of cell viability and IL-6 level in HIVIL treated with paradigm compounds. Dose-dependent decrease of cell viability (grey) and IL-6 level (black) were detected in TVX (a), CPZ (c), APAP (d), DIC (e), SLD (f), NVP (g) and PTU (h), but not in the negative analogue LVX (b). Data is presented as a percentage of LPS-treated vehicle control. Error bars represent s.e.m, *n* = 3. Two-way ANOVA was performed. * Denotes the significance in comparison to cell viability at respective concentrations, where *:*p <* 0.05, **:*p <* 0.01, ***:*p <* 0.001, ****:*p <* 0.0001.

IL-6 is known to plays a driving role in liver function homeostasis and hepatic inflammation (Jover et al., 2002), we further investigated the importance of drug-specific IL-6 decrease in HIVIL. Interestingly, we observed that changes in cytokine production occurred at much lower concentrations when compared to the IC_50_ of the drugs. The inability of existing models to capture the hepatotoxicity at concentrations close to physiological concentrations (e.g. 100-fold of Cmax was used in Xu et al., 2008)) motivated us to compare the performance of IL-6 and cell viability as endpoints (Fig. 5). Treatment with drugs caused a decline in IL-6 production at concentrations where majority of the cells were still viable. IC_50_ of IL-6 and cell viability inhibition by TVX was 16.2 μM and 87.7 μM, respectively (Fig. 5a). Similarly, IL-6 decline occurred at a lower dosage upon the treatment with CPZ, DIC, SLD, NVP or PTU when compared to changes in cell viability (Fig 5c–h) (Table S3). Treatment with LVX did not cause any change in cell viability and IL-6 production (Fig. 5b), indicating that only inflammation associated drugs cause changes in cell viability and IL-6. Overall, the data suggests that HIVIL allows recapitulation of cytokine changes at close to physiological concentrations as compared to conventional cell viability assays.

### 2.5 HIVIL model captures clinically relevant cytokine changes and hepatotoxicity *in vitro*

**Fig. 6:**
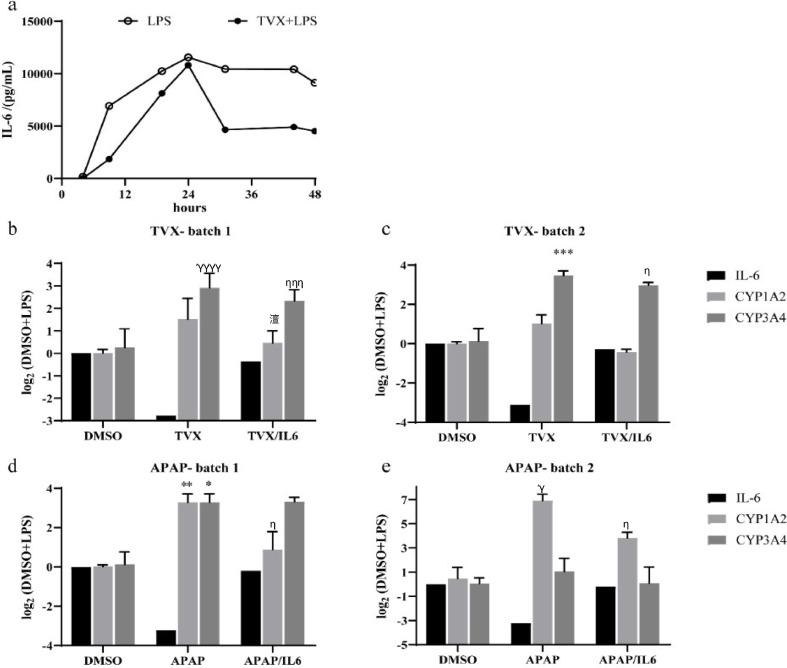
Decrease of IL-6 in HIVIL is associated with metabolic alteration. (a) IL-6 dynamics in HIVIL upon TVX treatment. (b-c) Transcriptional level of metabolic enzymes, cytochrome P450 3A4 (cyp3a4) and cyp1a2 was examined in 2 independent batches of HIVIL treated with TVX, with and without exogenous IL-6 (b-c). cyp1a2 expression was examined in 2 independent batches of APAP-treated HIVIL (d-e). Error bars of IL-6 are not indicated as data was presented as separate batches. Data is presented as the log2 fold to LPS-treated vehicle control. Error bars represent s.e.m, n = 3. One-way ANOVA was applied. *p < 0.05, **: *p <* 0.01 and ***: *p <* 0.001 between treatment and vehicle control. #: *p <* 0.05 between treatment and treatment with exogenous IL-6.

It is well known that increased IL-6 can inhibit the transcription and activity of several cytochrome P450 (CYP) enzymes (Ning et al., 2017). We investigated whether the reduction of IL-6 caused by drugs could reverse CYP suppression. In LPS-activated HIVIL, IL-6 production increased during the first 24 h, both with and without TVX treatment. After 24 h, IL-6 levels sharply declined by 57.3 % in cells co-treated with TVX and LPS, compared to only a 20.9 % decline with LPS alone (Fig. 6a). Thus, we supplemented recombinant IL-6 to TVX and LPS-treated HIVIL at 24 h to compensate for reduced IL-6 levels. TVX inhibited LPS-induced IL-6 production in HIVIL and simultaneously increased expression of CYP1A2 and CYP3A4 by 2.6-fold and 2.9-fold, respectively, exemplified in batch 1 (Fig. 6b). Supplementation with exogenous IL-6 reversed these CYP expression increases, suggesting IL-6-dependent dysregulation of hepatocellular metabolism by TVX (Fig. 6b-c). Similar effects were observed in two independent APAP-treated HIVIL batches, where CYP1A2 transcription was also IL-6-dependent (Fig. 6d-e). The results are presented separately due to variations in exogenous IL-6 calibration (Materials and Methods Section 5.3). Gene expression data from qPCR are summarized in Table S4. Overall, reduced IL-6 levels appear to regulate CYP expression and potentially affect drug metabolism in hepatocytes.

**Fig. 7:**
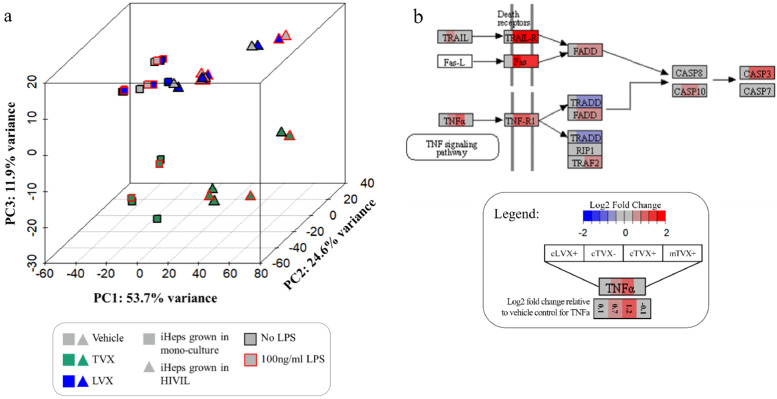
Transcriptomic analysis revealed alterations in cytokine-mediated apoptosis pathways as hepatotoxic mechanism of TVX in co-culture. (a) Principal component analysis plot of all sequenced HIVIL samples to examine the effect of drug treatment. RNA-Seq was conducted in a 2 × 3 × 2 full experiment design: iHeps grown in mono-culture vs. iHeps grown in co-culture (square vs. triangle), TVX vs. LVX vs. vehicle treatment (green, blue and grey symbols) and LPS presence vs. absence (black vs. red outline). (b) Subset of TNFα-related genes involved in apoptosis, with full KEGG apoptosis pathway shown in Fig. S4d.

To further investigate cytokines' roles in hepatotoxicity and the necessity of HIVIL components in recapitulating these mechanisms, RNA-Seq was performed on iHeps from HIVIL treated with TVX (25 µM) and its non-hepatotoxic analogue, LVX. Principal component analysis (PCA) showed HIVIL effectively distinguished TVX from non-DILI treatments (LVX and vehicle controls) along PC3 based on global transcriptomic profiles (Fig. 7a). Subsequent analyses compared gene fold-changes of treated samples against their respective vehicle controls: "m" and "c" indicate mono- and co-culture, and "–" and "+" represent the absence or presence of LPS. IL-6 transcription slightly decreased in iHeps treated with TVX regardless of culture conditions but remained unchanged after LVX treatment (Fig. S4a). IL-8 transcription increased by 1.3-fold in mono-culture (mTVX+ vs. mVeh+) and by 3.2-fold in co-culture (cTVX+ vs. cVeh+), consistent with increased MIP-2 (murine IL-8 equivalent) reported in TVX-treated mice (Fig. S4b) (Giustarini et al., 2019). TNFα, known to synergize with TVX-induced apoptosis and hepatotoxicity (Beggs et al., 2014; Shaw et al., 2007) was most notably elevated in cTVX+ (2.3-fold increase) (Fig. S4c). Consequently, two key TNFα-pathway effector genes, FADD and caspase 3, showed respective 1.7- and 1.8-fold upregulation in cTVX+ (Figs. 7b, S4d). Although mTVX+ similarly upregulated FADD and caspase 3, this increase was independent of TNFα (0.9-fold compared to mVeh+; Figs. 7b, S4d). Furthermore, other apoptosis-related factors, including p53, Fas, Bim, Bad, Bax, and cell cycle inhibitor p21, were also upregulated by TVX treatment (Fig. 7b). The TNFα-activated pro-survival NF-κB pathway, including apoptosis inhibitor cIAP-2, showed greater upregulation in cTVX+ (3-fold) compared to mTVX+ (1.5-fold) (Fig. 7b). Overall, our data shows that cytokine changes in co-cultures lead to changes in drug metabolism in hepatocytes which match reported cytokine changes associated with hepatotoxicity (including IL-6-mediated CYP regulation and TNFa-mediated pro-apoptosis and pro-survival regulation). However, this is only applicable for co-cultures where downstream effects can be traced back to cytokine changes, and not in monocultures where significant upstream cytokine changes are not observed.

### 2.6 HIVIL with THP-1 does not recapitulate clinical cytokine responses

THP-1 macrophages are commonly used as substitutes for primary human Kupffer cells (PHKCs) in modeling immune-mediated hepatotoxicity (Edling et al., 2009; Granitzny et al., 2017; Wewering et al., 2017). Thus, we assessed drug-specific cytokine effects in an HIVIL model using THP-1 macrophages co-cultured with iHeps. THP1- derived macrophages were obtained as reported elsewhere (Zeng et al., 2015). These THP-1 derived macrophages maintained their functionality in William E-based medium optimized for HIVIL compared to standard THP-1 medium. However, unlike iKCs, THP-1 macrophages failed to replicate IL-6 increases upon treatment with AQ, INH, LTG, and PHT, and did not detect the diclofenac (DIC)-specific IL-6 reduction (Fig S6). THP-1 cells showed poor sensitivity and specificity in mimicking drug-specific IL-6 responses, highlighting the necessity of Kupffer cells for accurate cytokine response modeling. Further analysis compared co-culture induced changes in HIVIL (cVeh+ vs. mVeh+) against an indirect co-culture of HepG2 and THP-1 macrophages (Padberg et al., 2020). KEGG pathway analysis revealed that differentially expressed genes unique to HIVIL predominantly involved inflammation-related pathways, notably cytokine-cytokine receptor interaction and NOD-like receptor signaling (Table S5). In contrast, Padberg et al., identified no major immune-related pathways except autophagy (Table S6), emphasizing the critical role of liver-specific macrophages in capturing appropriate immune responses.

## 3 Discussion

Immune-mediated responses play a critical role in drug-induced liver injury (DILI). This study proposes the use of induced pluripotent stem cell-derived Kupffer cells (iKCs) to capture drug-specific cytokine responses *in vitro*. The HIVIL model successfully recapitulated clinical cytokine responses from six paradigm compounds (AQ, DIC, INH, LTG, PHT, and TVX) and responses from ten additional *in vivo* training compounds. Changes in IL-6 production were mechanistically related to hepatocyte metabolism through regulation of CYP1A2 and CYP3A4. Importantly, cytokine changes observed with iKCs were not reproduced when substituted with THP-1 cells.

We present an updated version of our previously published iKC differentiation protocol, which significantly improves cell yield, function, and marker expression (Tasnim et al., 2019). Co-culture conditions with induced hepatocytes (iHeps) were optimized, enhancing hepatocyte performance and function in the HIVIL model. Notably, iHeps co-cultured with iKCs demonstrated improved hepatocyte function compared to iHeps alone or primary human hepatocytes (PHH) in collagen sandwich cultures (Xia et al., 2016).

More importantly, the drug toxicity IC_50_ response of HIVIL co-culture was similar to that reported in the state-of-the art, indicating sufficient functional and metabolic capacity of iHeps. For instance, IC_50_ of DIC in HIVIL model has been predicted to be 385 mM (Fig. 5), which corroborates with the IC_50_ reported by PHH-PHKC co-culture (IC_50_∼127 mM) (Messner et al., 2013; Sarkar et al., 2017), and is strikingly different from the reported mono-cultures to be about 1 mM in PHH (Mennecozzi et al., 2015) and iHeps mono-cultures (Kia et al., 2015). We compared drug-specific cytokine responses in HIVIL to respective clinical and *in vivo* responses. HIVIL accurately recapitulated cytokine changes observed clinically for AQ, INH, LTG, PHT (increased IL-6), and TVX (increased TNFα) (Fig. 3). Additional testing with compounds reporting *in vivo* cytokine responses also exhibited good *in vitro*-*in vivo* correlation, except for leflunomide (LFM) and sulfasalazine (SSZ) (Fig. 4b). The discrepancy with SSZ likely arises from the involvement of eosinophils and T cells in hypersensitivity responses, immune cells absent in HIVIL (Mennicke et al., 2009). Though clinical IL-6 changes have yet to be reported for chlorpromazine (CPZ), nevirapine (NVP), and propylthiouracil (PTU), our data suggest a probable IL-6 decrease in patients. Collectively, HIVIL showed 88.9 % (16/18) sensitivity in capturing drug-specific cytokine responses.

Both IMR90- and F4-based HIVIL models revealed that reduced IL-6 correlated with de-suppression of CYP1A2 and CYP3A4 upon acetaminophen (APAP) or TVX treatment (Figs. 6; S5e–h), potentially indicating drug metabolite accumulation. Mono-culture hepatocyte models fail to capture these immune-metabolic interactions, highlighting HIVIL’s potential to clarify drug-cytokine metabolic interactions. To elucidate hepatotoxicity mechanisms, RNA-Seq analyses were conducted in HIVIL configurations treated with TVX and levofloxacin (LVX). In mice, TVX is known to block neutrophil recruitment, increasing IL-8 and TNFα production, leading to liver injury via apoptosis (Giustarini et al., 2019). HIVIL, but not mono-cultures, exhibited increased IL-8 and TNFα transcription following TVX exposure (Fig. S4b-c). Additionally, apoptosis markers and caspase 3 transcription increased in both mono-culture and co-culture. However, TNFα signaling was exclusive to co-cultures, indicating cytokine-mediated apoptosis. Mono-cultures likely showed apoptosis through cytokine-independent pathways like DNA damage responses (Beggs et al., 2015). Thus, RNA-Seq confirmed HIVIL accurately recapitulates reported TVX-induced hepatotoxic mechanisms, whereas mono-cultures did not. This also helps to explain why no significant difference in cell viability was observed when cTVX+ and mTVX+ were compared (mTVX+: 53.7 % ±10.0 % (data not shown), cTVX+: 63.4±5.0 % (Fig. 5a)), further emphasizing our hypothesis that cell viability alone might not be a sensitive marker for predicting drug responses.

Finally, replacing iKCs with THP-1 cells in HIVIL resulted in distinct IL-6 profiles (Fig. S6), highlighting significant functional differences. Differential gene expression was predominantly immune-related, reinforcing iKCs’ superior suitability for modeling human immune-mediated drug responses compared to THP-1 (Lokhonina et al., 2019; Padberg et al., 2020). Therefore, iKCs are an excellent substitute to PHKCs for recapitulating human responses *in vitro* and could not be replaced with THP-1 or other peripheral monocytes-derived macrophages in a functional *in vitro* model. While iKCs play an important role in immune-mediated DILI, recent studies have additionally shown effects of neutrophils and circulating monocytes on immune response. It would be important to include these cells in the system in future studies in order to obtain a more complete understanding of the system and to improve predictivity of drug immune responses; however, it needs to be determined if the increase in sensitivity justifies the added complexity. Further, the model can be extended to incorporate HLA polymorphisms associated DILI by utilizing iPSCs from HLA polymorphic donors.

## 4 Conclusion

HIVIL comprising of iHeps and iKCs represents a minimal model to investigate drug-induced hepatotoxic response of drugs *in vitro.* HIVIL recapitulates clinical / *in vivo* drug effects under inflammatory conditions and can differentiate drugs based on their immune-mediated effects, which could not be achieved by co-culture of iHeps and THP-1. The model will be critical in screening immune-mediated effects of drugs and warranting further investigation of new drugs which cause cytokine imbalances.

## 5 Materials and methods

### 5.1 Cell culture and differentiation

Human iPSC cell line iPS (IMR90)-4 was purchased from WiCell Research Institute (Madison, WI), and maintained with a feeder-free method according to the instruction from STEMCELL Technologies (Vancouver, BC, Canada). The protocol of differentiation towards iKCs and iHeps as described previously with slight modification (Tasnim et al., 2019; Cai et al., 2012), respectively. The re-optimized protocol to generate iKCs is discussed in Results section. More details are provided in Supplementary Methods. After differentiation, iKCs was harvested using 0.05 % trypsin-EDTA (Gibco, Waltham, Massachusetts, USA), while iHeps were dissociated with TrypLE^TM^ (Gibco). iHep suspension was further filtered through a 40 µm cell strainer to remove extracellular matrix clumps. Cell suspension was centrifuged at 1000 rpm for 5 min. For co-culture set up, tissue culture plates were coated with Rat tail collagen I (Gibco) diluted 100-fold in 0.02 N acetic acid, and incubated at 37 °C for 1 h and washed 3 times with PBS. According to the protocol optimized by Tasnim et al. (2019), iKCs was firstly seeded at a density of 14,000 per well on 96-well plate with Advanced DMEM (plus supplements) containing 5 % FBS. On the following day, iHeps were added at a density of 35,000 per well in Williams’ Medium E (plus supplements) (iHep: iKCs ratio = 2.5: 1). This reflects the physiological ratio of these cells *in vivo* under inflammatory conditions (Tasnim et al., 2021). Fresh media containing drugs was applied to co-cultures the next day. 50,000 cells were seeded to establish iHeps monoculture. THP-1 cells were kindly obtained from Dr. Eliza Fong and cultured as described by Zeng et al. (2015). Details provided in Supplementary Methods.

### 5.2 Drug administration

After iHeps were seeded for 24 hr, HIVIL was subjected to treatments including 100 ng/ml LPS (Sigma Aldrich, St. Louis, USA), IL-2 (R&D Systems) and drugs (Sigma Aldrich) (with/without LPS) for 48 hr. Stock solutions of drugs were prepared in dimethyl sulfoxide (DMSO) and dissolved in medium prior to use. Medium containing 0.1 % DMSO was used as vehicle control except for APAP and PZA treatment. For these two drugs, the highest DMSO concentration was 1 %. Supernatant was collected at 48 hr for measurement of cytokines, and remaining cells were subjected to viability assay prior to lysis for RNA extraction.

### 5.3 Exogenous IL-6 addition and calibration

The absolute level of IL-6 produced from LPS-induced HIVIL cultured on 96-well plate ranged from 1000 to 10,000 pg/mL (data not shown). To compensate the reduced exogenous IL-6 without significantly changing the total volume, Recombinant human IL-6 (R&D Systems) was reconstituted and diluted with the same medium used for HIVIL culture and added to drug-treated wells as 100x concentrated solution. To start with, to a 500 ng/mL (#0) solution was prepared. Additional 2-fold serial dilution of #0 was prepared and added (1:100) to HIVIL that had been treated with TVX or APAP for 24 hr. PCR analysis was conducted on samples showing the same IL-6 level as DMSO+LPS control.

### 5.4 RNA-Seq analysis

RNA extraction was done using TRIzol and analyzed on a Tapestation 4200 to determine RNA Integrity Number (RIN). Only samples with RIN > 8 were sequenced. Illumina Stranded mRNA multiplexing was done with unique 96 dual indices, and all samples sequenced on the same lane of NovaSeq 6000 S4 flowcell. Data normalization was performed using DESeq2 v1.32.0 (Love et al., 2014) on the R programming language v4.1.0 (R Core Team, 2021; R Team, 2021). Gene enrichment analysis was done with fgsea v1.18 (Korotkevich et al., 2021) with gene sets downloaded from MSigDB and KEGG pathway visualization done via pathview v1.32 (Kanehisa et al., 2021; Luo and Brouwer, 2013). Other plots were produced using ggplot2 v3.3.5 and scatterplot3d v0.3-41 (Ligges and Maechler, 2003). The RNA-Seq dataset has been deposited in NCBI under accession number GSE189320.

Further details on RNA-Seq analysis and common assays including cell viability, ELISA, qPCR, albumin /urea measurement and CYP activity are provided in Supplementary methods.

### 5.5 Statistical analysis

Mean and standard deviation were obtained from at least three independent batches of cells. Unpaired, paired student’s t-test and one-way or two-way analysis of variance (ANOVA) were performed accordingly at an overall confidence level of 95 % using Prism software (GraphPad, San Diego, CA, USA) and indicated below each figure.

## CRediT authorship contribution statement

**Xiaozhong Huang:** Writing – review & editing, Writing – original draft, Validation, Methodology, Investigation, Formal analysis, Data curation. **Yun Ting Soong:** Visualization, Project administration, Investigation. **Jiahao Wang:** Visualization, Software, Data curation. **Claire Jia Yi Ng:** Methodology, Investigation. **Kartik Mitra:** Writing – review & editing. **Farah Tasnim:** Writing – review & editing, Writing – original draft, Validation, Supervision, Project administration, Formal analysis. **Hanry Yu:** Writing – review & editing, Supervision, Project administration, Funding acquisition, Conceptualization.

## Declaration of competing interest

The authors declare the following financial interests/personal relationships which may be considered as potential competing interests:

Hanry yu has patent Methods of generating hepatic macrophages and uses thereof pending to Agency for Science Technology and Research Singapore. Hanry Yu reports a relationship with Ants Innovate Pte Ltd that includes board membership and equity or stocks. Other authors have no competing conflict. If there are other authors, they declare that they have no known competing financial interests or personal relationships that could have appeared to influence the work reported in this paper.

## Data Availability

Data will be made available on request.

## Appendix Supplementary materials

Image, application 1
