# Supplementary material for: HIVIL: A human in vitro inflammatory liver model recapitulates immune-associated drug effects with high predictivity
Source: NAM J. 2025 Jun 24;1:100032. doi: 10.1016/j.namjnl.2025.100032 (PMC13289212; doi:10.1016/j.namjnl.2025.100032)
Supplement: Supplementary file 1 [file mmc1.docx]

**HIVIL: A Human *In vitro* Inflammatory Liver Model Recapitulates Immune-associated Drug effects with High Predictivity**

Xiaozhong Huang ^1,3^, Yun Ting Soong^1^, Jiahao Wang^5^, Claire Jia Yi Ng^1^, Kartik Mitra ^3^, Farah Tasnim^1,2^ and Hanry Yu ^3,4,5, *^

1. Institute of Bioengineering and Bioimaging, #07-01, 31 Biopolis Way, The Nanos, Singapore 138669, Singapore

2. Biomedical Sciences Industry Partnership Office (BMSIPO), A∗STAR, 31 Biopolis Way, Singapore 138669, Singapore

3. Department of Physiology, The Institute of Digital Medicine (WisDM), Yong Loo Lin School of Medicine, National University of Singapore, MD9-04-11, 2 Medical Drive, Singapore 117593, Singapore

4. CAMP, Singapore-MIT Alliance for Research and Technology, 1 CREATE Way, Level 4 Enterprise Wing, Singapore 138602, Singapore

5. Mechanobiology Institute, National University of Singapore, T-Lab, #05-01, 5A Engineering Drive 1, Singapore 117411, Singapore

* Co-corresponding author

Hanry Yu: phsyuh@nus.edu.sg, Tel: +65-65161644.

# Supplementary Figures and Tables Referenced in Main Text


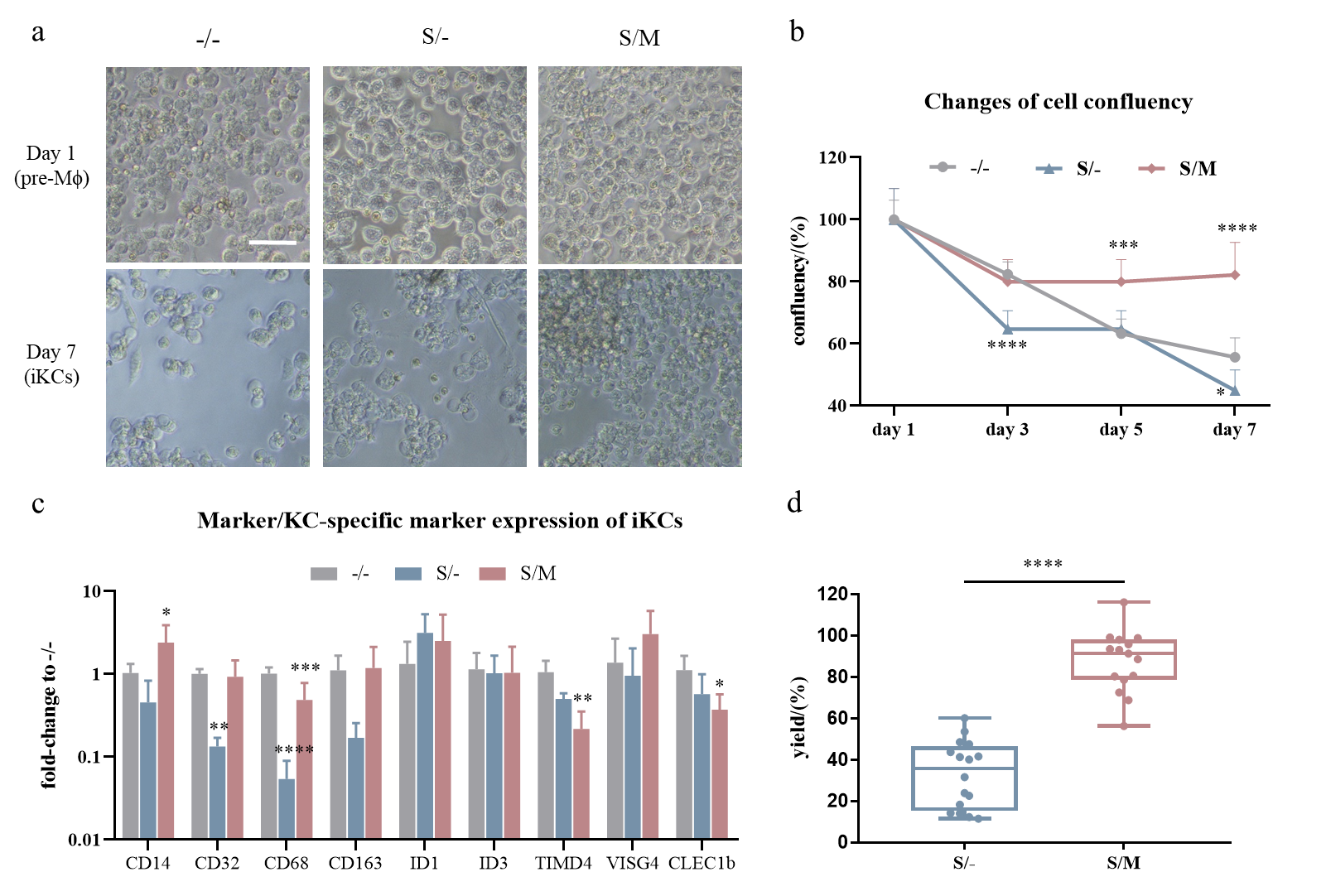


**Fig. S1** Optimization of culture conditions for improvement of iKCs yield. The use of S or M refers to the inclusion of serum or M-CSF in the course of differentiation respectively. (a) Cell confluency in various culture conditions. -/- refers to absence of both serum and M-CSF; S/- refers to addition of serum but no M-CSF (the original protocol by Tasnim *et al.*(Tasnim et al. 2019)); S/M refers to addition of both serum and M-CSF. scale bar: 100 μm. (b) Cell confluency across the 7-day differentiation period. Two-way ANOVA was performed. * indicates the significance of difference between tested conditions and -/- at particular time point: *: p<0.05, **: p<0.01, ****: p<0.0001. (c) Comparison of gene expression of key macrophage and KC-specific markers by qPCR. Relative expression of the tested genes to GAPDH was further normalized to -/-. Two-way ANOVA was performed. * denotes the significance in comparison to -/- for various genes tested, *: p<0.05, ***: p<0.001. (d) Yield comparison across 20 batches between S/- (original protocol) and S/M (re-optimized protocol). Yield % refers to cell number obtained on Day 7 as a percentage of that on Day 1. Student’s t-test was performed. ****: p<0.0001. Error bars represent s.e.m, n = 3

We further optimized our previous method of generating iKCs (Tasnim et al. 2019) by systematic characterization of serum and growth factor supplementation in order to improve cell yield. Instead of relying solely on serum for cell viability and attachment (Tasnim et al. 2019) (Fig. S1a, S/- panel), we tested the effect of macrophage colony-stimulating factor (M-CSF) due to its role in the development and maturation of KCs (Radi et al. 2011; Yamamoto et al. 2008). The addition of M-CSF could better maintain cell density on Day 7 than cultures without M-CSF (Fig. S1a, S/M panel), and resulted in an increase in cell number (Fig. S1b), regardless of the presence of serum. Additionally, M-CSF either stabilized or further improved the expression of macrophage and KC-specific markers (Fig. S1c). Since serum is important for initial attachment, and condition S/M could improve cell yield without compromising cell functions, we decided to use this condition for the subsequent experiments. Additionally, multiple batches of differentiation under S/- (the original protocol) and S/M (re-optimized protocol) showed approximately 2.67-fold increase in differentiation yield (32.73±16.19% vs 87.38±14.81%) upon M-CSF addition (Fig. S1d), which is in agreement with our density comparison (Fig. S1b). Collectively, these data demonstrate the benefit of M-CSF addition on iKC differentiation and yield.


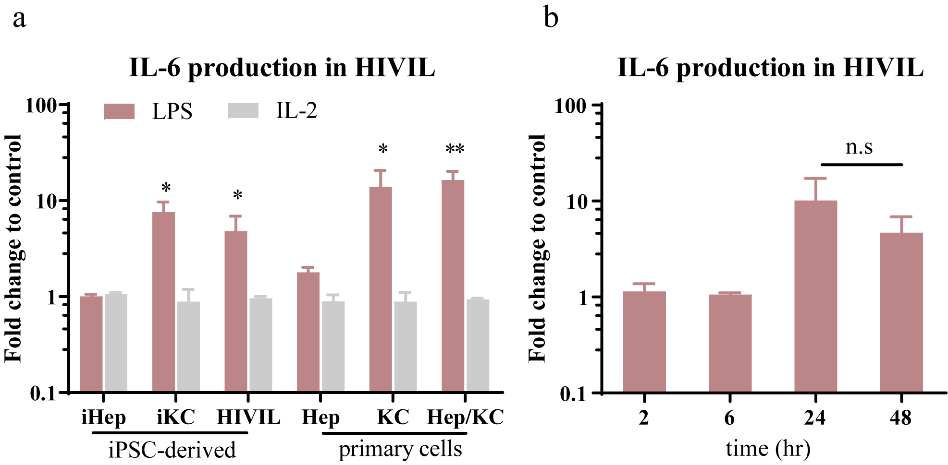


**Fig. S2** Optimization of stimulator and duration of stimulation. **(a)** Stimulating effect of lipopolysaccharide (LPS) or interleukin-2 (IL-2) in hepatocytes (Hep), KCs and HIVIL derived from human iPSC or primary cells. Data is presented as fold difference to unstimulated control. Student’s t-test was used. *: p<0.05, **: p<0.01. asterisks represent comparison to cytokine production in unstimulated control. (b) Time-dependent IL-6 production upon LPS stimulation. Student’s t-test was performed. ns: non-significant., *: p<0.05, **: p<0.01;

Neither LPS nor IL-2 stimulated IL-6 production from PHHs and iHeps (Fig. 1g). LPS, but not IL-2, stimulated IL-6 production in iKCs and PHKCs, in mono-culture and co-culture with iHeps (Fig. S2a). Very low basal levels of IL-6 were observed for unstimulated controls and for stimulated iHeps cultures (data not shown as absolute values as cytokine levels were normalized to vehicle + LPS control for standardization). These findings are in line with Nguyen *et al.* (Nguyen et al. 2015), suggesting the inability of human KCs *in vitro* to respond to IL-2. Therefore, LPS was used for subsequent experiments to stimulate inflammation. Although LPS has been widely used, duration of LPS stimulation varies greatly in different studies (Bonzo et al. 2015; Nguyen et al. 2015; Rafal P. Witek and Cornelia Smith 2010; Rose et al. 2016). In order to evaluate the time-dependent inflammatory responses, we measured interleukin-6 (IL-6) production within 48 hr post-LPS treatment. IL-6 was only produced at 24 hr and 48 hr, by 10.15- and 4.70-fold compared to vehicle control respectively, but not at 2 hr and 6 hr (Fig. S2b). The difference between 24 hr and 48 hr was non-significant (p=0.41), indicating IL-6 response plateaus after 24 hr. Considering the possibility of synergistic effects of LPS and drugs between 24 hr and 48 hr and the observed desensitization of KCs to LPS after chronic exposure (decrease in cytokine levels after 48 hours (Rafal P. Witek and Cornelia Smith 2010)), we decided to treat the cells for 48 hr.

We also reviewed the state-of-the-art to investigate the challenges of generating robust *in vitro* data which have good *in vivo* correlation. Two factors that vary quite greatly in different studies are the LPS and drug concentrations. Commonly used LPS concentrations span across a wide range: 0.1 - 10 μg/mL, with the clinically relevant LPS concentration between 0.1 - 10 ng/mL (Guo et al. 2013). In order to use closer-to-physiological range concentration in our HIVIL model, we took the midpoint of this range (5 ng/mL) and used 20x median physiological concentration (100 ng/mL). RNA-Seq confirmed that this choice did not overstimulate the iHeps as almost no changes in gene expression were observed with LPS-only treatment. The large differences in LPS concentration between our study and others might cause discrepancies in drug-induced cytokine effects. For instance, immune-mediated response to INH was correctly classified in our model; however, INH did not show any changes in IL-6 levels even at 858.6 μM when 1 μg/mL of LPS was used in a previous study (Li et al. 2020). Additionally, IL-6 decreased upon APAP treatment in our model (similar to *in vivo* findings), whereas it increased in other *in vitro* models deploying 1-10 μg/mL of LPS (Jiang et al. 2019; Li et al. 2020). This highlights 1) the value-add of our model and 2) the need to optimize and standardize culture and treatment conditions for *in vitro* models


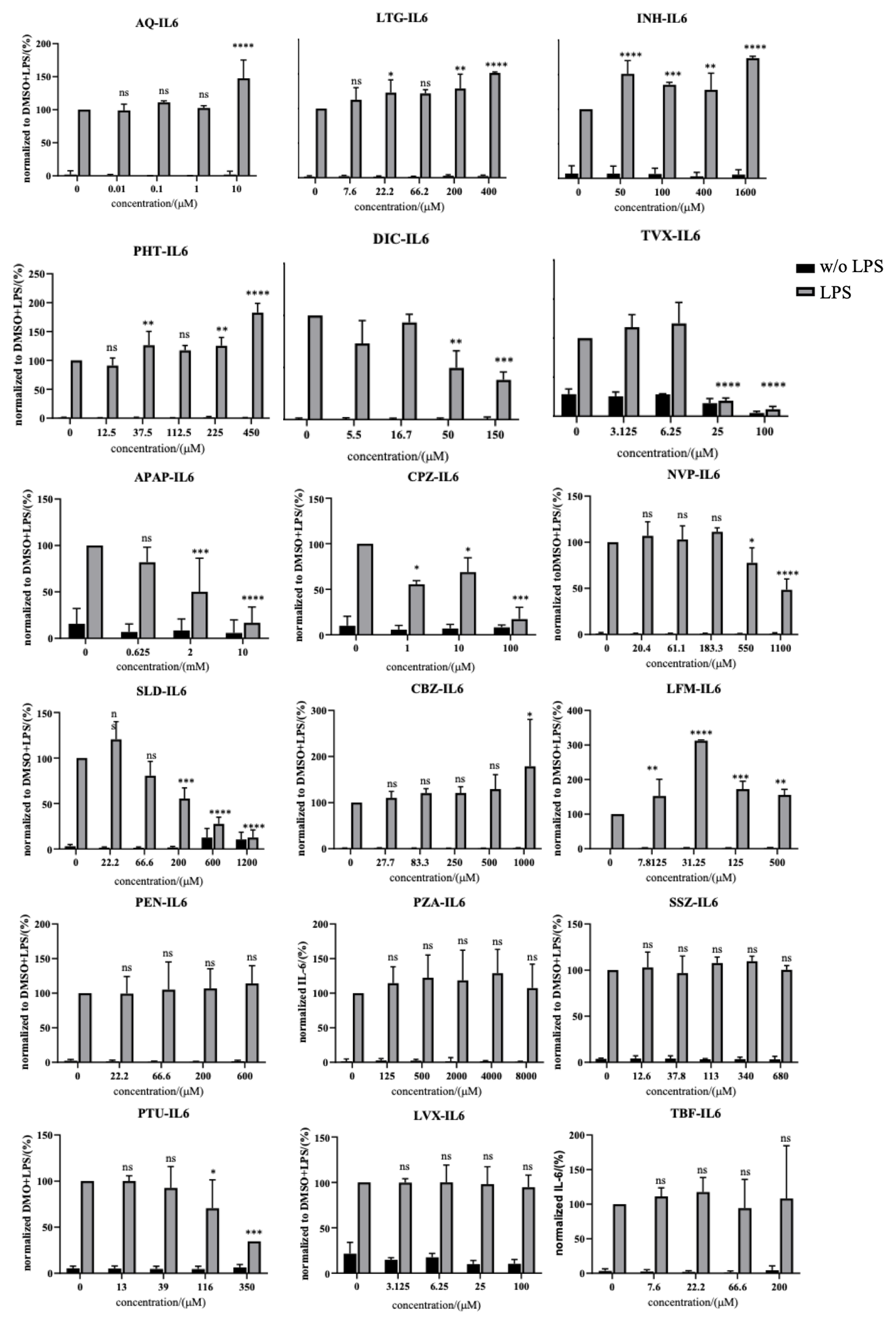


**Fig. S3** IL-6 response of known DILI agents in the presence and absence of LPS.


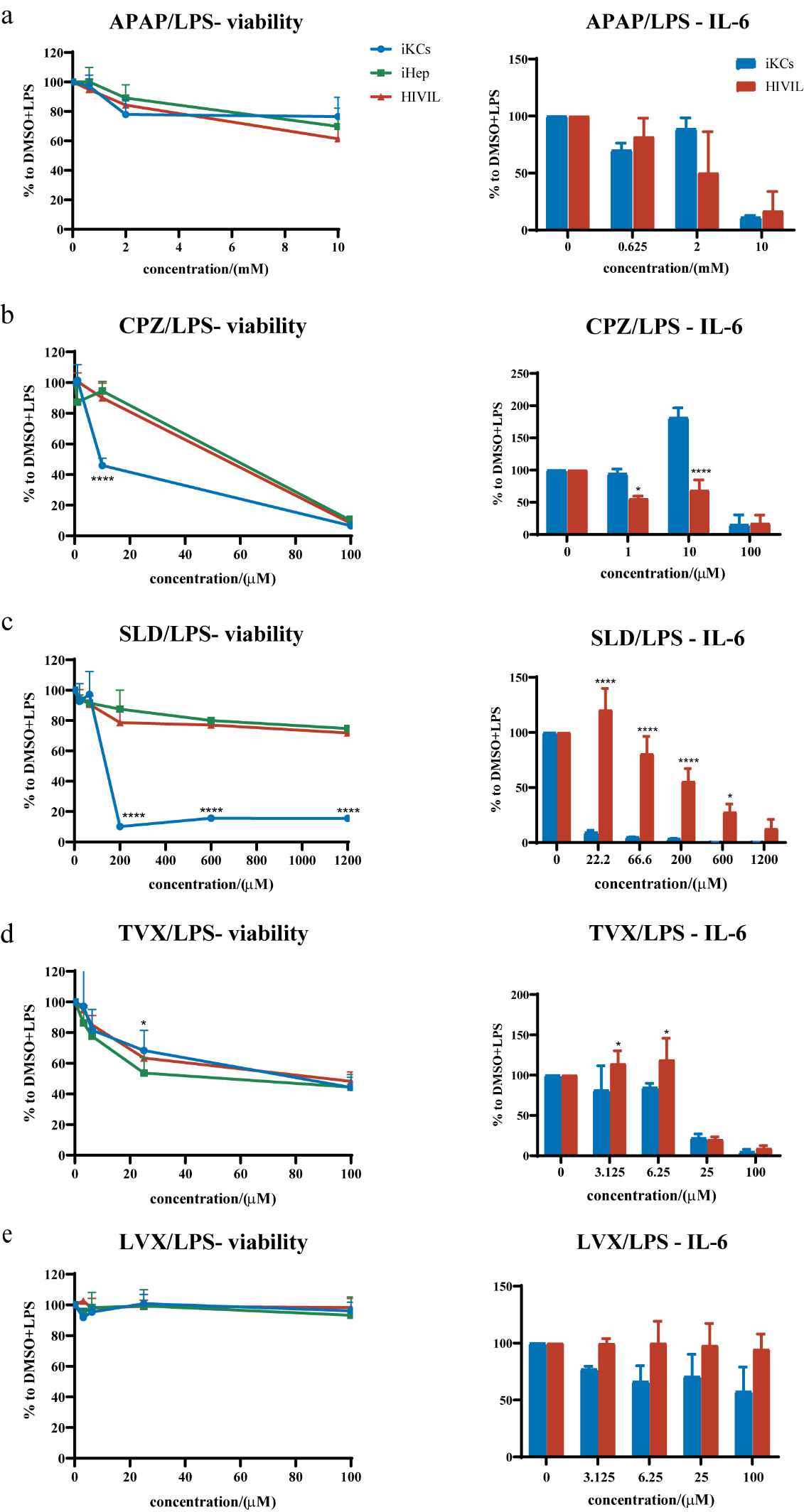


**Fig. S4** Cytotoxic and inflammatory effects of APAP (a), CPZ (b), SLD (c), TVX (d) and LVX (e) on HIVIL (red) and mono-cultures of iKCs (blue) and iHep (green). Cytokine production is normalized to cell viability and presented as the percentage of the LPS-treated vehicle control. Two-way ANOVA was applied. *indicates significance in comparison to iHep mono-culture, where *:p<0.05, ****:p<0.0001. Cytokine production from iHep mono-culture is negligible and is not displayed in the cytokine panel. Cytokine levels are displayed as percentage to DMSO + LPS control for normalization. Statistical analysis was performed using one-way ANOVA, **:p<0.01, ***:p<0.001, ****:p<0.0001.

The viability profiles were similar among iHeps, iKCs and HIVIL when treated with APAP, TVX or LVX, suggesting that any decrease in cytokine production upon treatment with these drugs cannot be attributed to overt toxicity to iKCs. The only exception was SLD, which caused substantially higher toxicity to iKCs mono-culture compared to iHeps mono-culture and HIVIL. Because of these cytotoxic effects, the cytokine production from iKC mono-culture was abolished in SLD-treated cultures. Treatment with 10 mM CPZ caused a 50% decrease in viability in iKCs, however IL-6 production was still higher than that in the HIVIL model. Despite no differences in cell viability between iKCs and HIVIL upon APAP treatment, 2 mM APAP treatment caused a reduction of IL-6 in HIVIL by almost 50%, while IL-6 levels in iKC mono-culture remained unchanged at this concentration. Similarly, LVX-specific IL-6 responses were also different between iKCs mono-culture and HIVIL. This suggests that changes in cytokine levels in HIVIL are not caused by effects of the drugs on iKCs alone but are also affected by the differential effects of parent compounds and processed products metabolized by hepatocytes. Hence, both cell types (iKCs for their cytokine-producing ability and iHeps for their drug metabolism activity) are critical for mimicking immune-mediated effects of drugs in HIVIL.


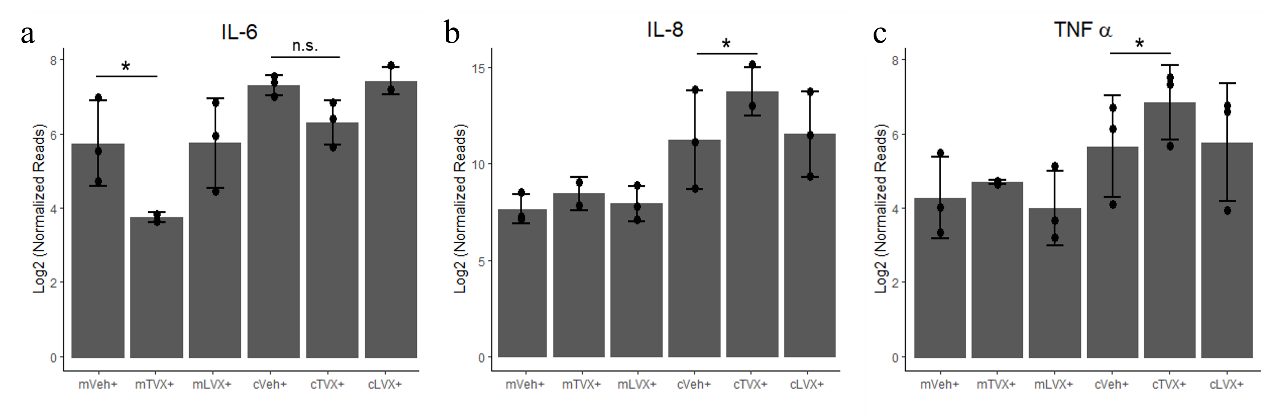


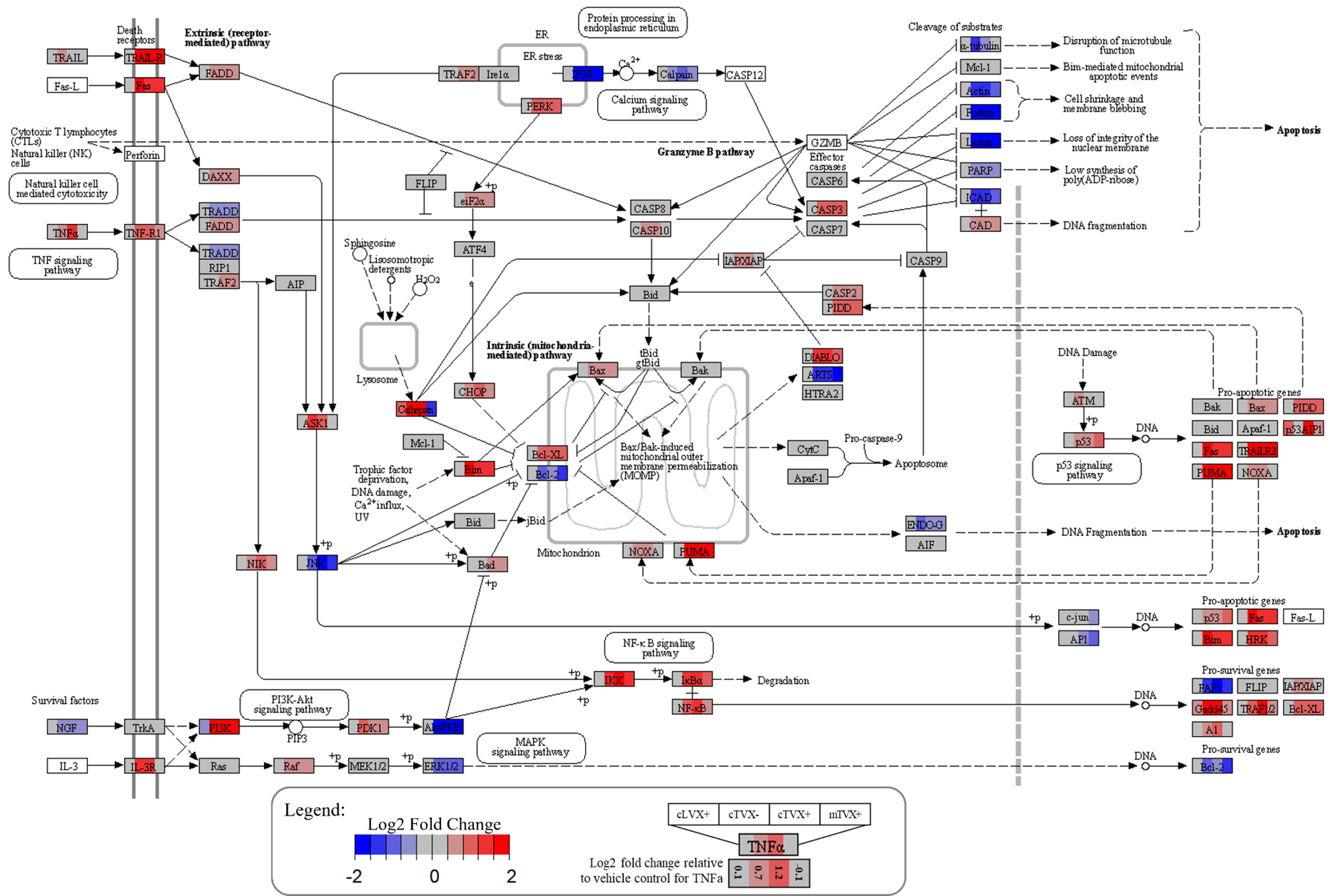


d

**Fig. S5** (a-c) Transcriptional changes of key cytokines measured using RNA-Seq. The bar heights represent the mean of log2 (normalized read counts) with standard deviation plotted as error bars. Samples were named as: <mono-or co-culture (m or c)> <treatment (Veh or TVX or LVX)> <absent or including LPS (- or +)>. E.g., mTVX+ indicates an iHep mono-culture treated with TVX and LPS. Tests for statistical significance between drug-treated samples and their respective vehicle control was carried out in DESeq2, with multiple test-adjusted p-value = 0.05 as the threshold. (d) Please view the online version for full-sized image in color. The color of the block indicates the log2 fold change of the gene in the drug treatment relative to its vehicle control, with the gene encoding TNFα illustrated in the legend box as an example. A white box indicates the gene transcript is not present in the dataset. Samples were named as: <mono-or co-culture (m or c)> <treatment (Veh or TVX or LVX)> <absent or including LPS (- or +)>. E.g., mTVX+ indicates an iHep mono-culture treated with TVX and LPS. cLVX+ was included to highlight the specificity of HIVIL with a non-hepatotoxicant. To showcase smaller gene expression changes, the highest absolute log2 fold changes shown in the diagram was capped at 2, i.e., the change *may* be larger than 4-fold, especially in effector genes. The cap was drawn to emphasize the direction of change rather than magnitude.


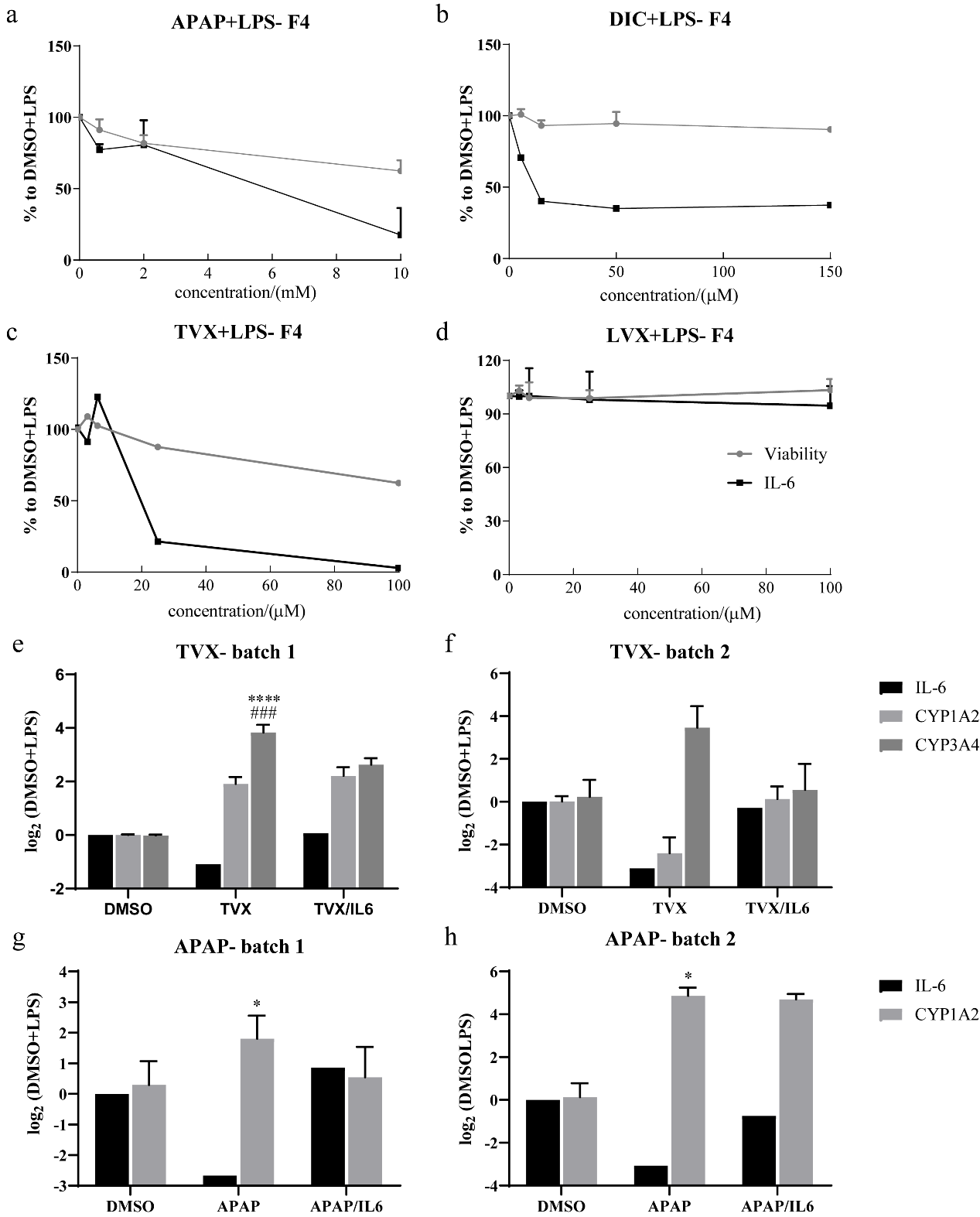


**Fig. S6 Decrease of IL-6 in iPSC(F4)-derived HIVIL is associated with metabolic alteration.** HIVIL exhibited similar dynamics of cell viability and IL-6 when treated with (a) TVX, (b) APAP and (c) LVX when iPSC(F4)-derived cells were used. Decreases in IL-6 resulted in de-repression of cyp3a4 and cyp1a2 in TVX (e-f), as well as cyp1a2 in APAP, as examined by 2 independent batches (g-h). Data is presented as the fold difference to LPS-contained vehicle control. Error bars represented S.D. from at least 2 technical repeats. One-way ANOVA was applied. ***: p<0.05, and ****: p<0.0001 between treatment and vehicle control. ###: p<0.001 between treatment and treatment with exogenous IL-6.

We also developed HIVIL from another human iPSC cell line iPSC(F4), a kind gift from Dr. TAN Min-Han, to evaluate the model performance with APAP, DIC and TVX. Dose-dependent decrease in IL-6 production was observed in iPSC(F4)-derived HIVIL when treated with either APAP or DIC, which occurred at a lower concentration than the decrease in cell viability (Fig. S5a-b). 6.25 μM TVX introduced 1.2-fold increase in IL-6, while higher concentrations inhibited 80% and 97% of IL-6 production (Supplementary Fig. S5c). The negative compound LVX did not influence cell viability or IL-6 production (Supplementary Fig. S2d). Such IL-6 decline caused by TVX and APAP treatment was associated with the induced transcriptional level of CYPs, which would be repressed by the addition of exogenous IL-6 (Fig. S5e-h; Table S7). Collectively, the paradigm compounds TVX and APAP caused metabolic and inflammatory effects on HIVIL derived from iPSC(F4), with similar behavior as the iPSC (IMR90)-based HIVIL, indicating that HIVIL is potentially capable of recapitulating inflammation-associated DILI in iPSCs from different sources.


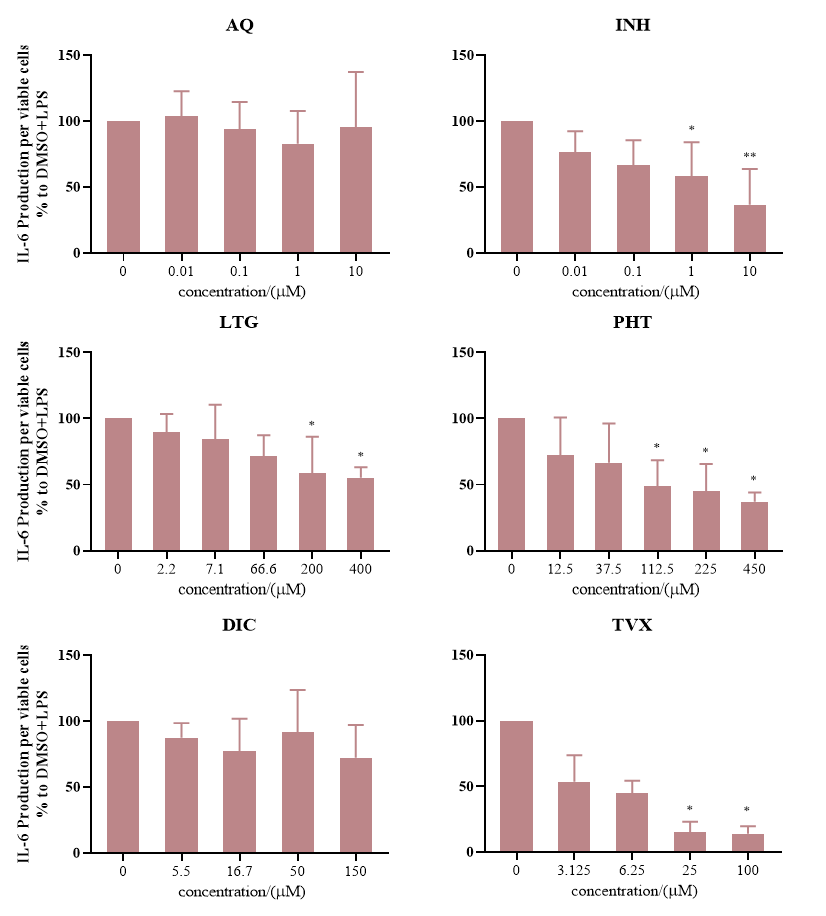


**Fig. S6** IL-6 responses from HIVIL deploying THP-1 macrophages upon treatment of training compounds: Amodiaquine (AQ), isoniazid (INH), phenytoin (PHT), diclofenac (DIC), sulindac (SLD) and trovafloxacin (TVX). Cytokine production was normalized to cell viability. Data is expressed as the percentage of the LPS-treated vehicle control. Error bars represent s.e.m, n = 3. One-way ANOVA was applied. *: p<0.05, **: p<0.01 between treatment and vehicle control.

|  | KEGG Pathway | padj | ES | NES | Size | Count | Leading Edge subset |
| --- | --- | --- | --- | --- | --- | --- | --- |
| 1 | Chemokine signaling pathway | 1.44E-14 | 0.66 | 2.44 | 163 | 46 | CCL24, CCL22, CCR5, PIK3R5, CCL3 |
| 2 | Cell adhesion molecules | 4.22E-14 | 0.70 | 2.48 | 118 | 37 | HLA-DRA, HLA-DRB1, HLA-DRB5, HLA-DQA2, CD86 |
| 3 | Cytokine-cytokine receptor interaction | 4.98E-11 | 0.61 | 2.25 | 183 | 75 | CCL24, TNFRSF14, CCL22, CCR5, CCL3 |
| 4 | Natural killer cell mediated cytotoxicity | 4.98E-11 | 0.70 | 2.43 | 95 | 27 | TYROBP, SYK, FCER1G, ITGB2, LCP2 |
| 5 | Intestinal immune network for IgA production | 3.16E-10 | 0.85 | 2.47 | 34 | 23 | HLA-DRA, HLA-DRB1, HLA-DRB5, HLA-DQA2, CD86 |
| 6 | Antigen processing and presentation | 1.26E-09 | 0.76 | 2.39 | 56 | 17 | HLA-DRA, HLA-DRB1, HLA-DRB5, HLA-DQA2, CTSS |
| 7 | B cell receptor signaling pathway | 2.44E-09 | 0.72 | 2.39 | 70 | 20 | SYK, FCGR2B, PIK3R5, BTK, VAV1 |
| 8 | Fc gamma R-mediated phagocytosis | 2.55E-09 | 0.67 | 2.31 | 94 | 24 | SYK, FCGR2B, PIK3R5, DOCK2, FCGR2A |
| 9 | Hematopoietic cell lineage | 3.00E-09 | 0.75 | 2.39 | 60 | 26 | HLA-DRA, HLA-DRB1, HLA-DRB5, CSF1R, ITGAM |
| 10 | Toll-like receptor signaling pathway | 1.01E-07 | 0.66 | 2.26 | 86 | 25 | CD86, IRF5, PIK3R5, CCL3, CCL4 |
| 11 | Leukocyte transendothelial migration | 2.91E-07 | 0.62 | 2.17 | 108 | 45 | NCF4, ITGB2, PIK3R5, PECAM1, ITGAM |
| 12 | NOD-like receptor signaling pathway | 1.27E-06 | 0.69 | 2.21 | 60 | 23 | NLRC4, CXCL8, PSTPIP1, NLRP3, PYCARD |
| 13 | Fc epsilon RI signaling pathway | 9.94E-06 | 0.64 | 2.11 | 69 | 19 | SYK, FCER1G, LCP2, PIK3R5, BTK |
| 14 | T cell receptor signaling pathway | 3.79E-05 | 0.58 | 2.00 | 94 | 19 | LCP2, PIK3R5, PTPRC, VAV1, IL10 |
| 15 | Steroid hormone biosynthesis | 9.43E-05 | -0.64 | -2.10 | 35 | 14 | HSD3B2, CYP1A1, CYP21A2, HSD17B2, UGT1A6 |
| 16 | Cytosolic DNA-sensing pathway | 1.27E-04 | 0.70 | 2.06 | 40 | 20 | CCL4, PYCARD, CCL4L2, CASP1, CCL5 |
| 17 | Lysosome | 3.19E-04 | 0.52 | 1.84 | 119 | 40 | ACP5, CTSS, LAPTM5, ATP6V0D2, SLC11A1 |
| 18 | Oxidative phosphorylation | 5.28E-04 | 0.51 | 1.82 | 119 | 72 | ATP6V0D2, NDUFA7, ATP6V0E2, ATP12A, UQCRHL |
| 19 | Regulation of actin cytoskeleton | 3.36E-03 | 0.44 | 1.63 | 200 | 53 | ITGAD, NCKAP1L, ITGB2, PIK3R5, ITGAX |
| 20 | Ribosome | 3.70E-03 | 0.52 | 1.76 | 85 | 70 | RPS15, RPL17, RPS28, RPS17, UBA52 |
| 21 | RIG-I-like receptor signaling pathway | 0.01 | 0.56 | 1.76 | 54 | 21 | CXCL8, DHX58, TNF, NFKBIA, STING1 |
| 22 | Complement and coagulation cascades | 0.01 | 0.54 | 1.78 | 64 | 18 | C1QC, C1QB, C1QA, C5AR1, F13A1 |
| 23 | Proteasome | 0.01 | 0.60 | 1.78 | 41 | 31 | PSMB9, PSMB8, PSMB10, PSME2, PSMD2 |
| 24 | JAK-STAT signaling pathway | 0.02 | 0.45 | 1.58 | 105 | 21 | PIK3R5, IL10RA, CSF2RB, IL10, IL12RB1 |

**Supplementary Table S1 KEGG pathways enriched in cVeh- relative to mVeh-.** Disease-specific pathways were excluded. NES refers to the normalized enrichment score. Size refers to the number of genes in the pathway and expressed in the sample. Count refers to the number of genes in the leading edge driving the pathway enrichment. Leading edge highlights the top 5 genes driving the pathway enrichment.

| Drug | Cmax/  (μM) | DILIrank (Chen et al. 2016) | DILIst (Thakkar et al. 2020) | Known immune-mediated effects (Ho et al. 2015; Kaplowitz 2005; Verma and Kaplowitz 2009) | Reported human responses | Reporte*d in-vivo* (murine) responses |
| --- | --- | --- | --- | --- | --- | --- |
| AQ | 0.1 (Ademisoye et al. 2018) | / | 1 | ✓ | IL-6↑ (Steuerwald et al. 2013) |  |
| DIC | 8.0 (Xu et al. 2008) | most | 1 | ✓ | IL-6↓ (Mahdy et al. 2002)  IL-10↑ (Mahdy et al. 2002) |  |
| INH | 76.6 (Xu et al. 2008) | most | 1 | ✓ | IL-6↑ (Steuerwald et al. 2013) | IL-6↑ (Hassan et al. 2016) |
| LTG | 9.8 (Agabeyoglu and Incecayir 2009) | most | 1 | ✓ | IL-6↑(Steuerwald et al. 2013) |  |
| TVX | 5.0 (Xu et al. 2008) | most | 1 | ✓ |  | TNFα↑ (Shaw et al. 2010)  IL-6↑ (Giustarini et al. 2019) |
| PHT | 15.4 (Rojanasthien et al. 2007) | most | 1 | ✓ | IL-6↑ (Steuerwald et al. 2013) |  |
| APAP | 138.9 (Xu et al. 2008) | most | 1 | ✓ |  | IL-6/IL-10 are protective factors (Ganey et al. 2004; Honarmand et al. 2012)  IL-10↓ (Bekker et al. 2016) |
| CBZ | 25.3 (Reunanen et al. 1992) | most | 1 | ✓ |  | IL-6↑(Higuchi et al. 2012) |
| LFM | 24.8 (Li et al. 2002) | most | 1 | ✓ |  | cytokines↓ via NF-kB (Manna and Aggarwal 1999; Yao et al. 2004) |
| SLD | 32.0 (Xu et al. 2008) | most | 1 | ✓ | mechanistic IL-6↓ (Kang et al. 2001) | TNFα↑ (Zou et al. 2009) |
| SSZ | 17.6 (Hong-mei et al. 2013) | most | 1 | ✓ | NF-κB inhibitor (Wahl et al. 1998) | IL-6↓ (Dik et al. 2018) |
| LVX | / | most | 1 | × |  |  |
| PEN | / | less | 1 | × |  |  |
| PZA | 406.9 (Bareggi et al. 1987) | less | 1 | × |  |  |
| TBF | 3.4 (Jiang et al. 2008) | most | 1 | × |  |  |
| NVP | 26.3 (Cammett et al. 2009) | most | 1 | ✓ |  | TNFα↑ (Bekker et al. 2012) |
| CPZ | 0.8 (Xu et al. 2008) | less | 1 | ✓ |  | TNFα↑ (Gandhi et al. 2013) |
| PTU | 17.6 (Mendes et al. 2014) | most | 1 | ✓ |  | TNFα↑ (Heidari et al. 2019) |

**Supplementary Table S2** Classification and inflammatory properties of the drugs. DILIrank: FDA-labeling information. DILIst: Drug-induced liver injury severity and toxicity: a binary classification of 1279 drugs by human hepatotoxicity.

| Compounds | IC_50_: IL-6 (μM) | IC_50_: viability (μM) | Cmax (μM) |
| --- | --- | --- | --- |
| APAP | 5358.3 | 10,170.3 | 138.9 (Xu et al. 2008) |
| CPZ | 12.9 | 34.4 | 0.8 (Xu et al. 2008) |
| DIC | 112.1 | 385.2 | 8.0 (Xu et al. 2008) |
| SLD | 380.7 | 2059.2 | 32.0 (Xu et al. 2008) |
| TVX | 16.2 | 87.7 | 5.0 (Xu et al. 2008) |
| NVP | 1071.4 | 2606.8 | 26.3 (Cammett et al. 2009) |
| PTU | 259.0 | N.A. | 17.6 (Mendes et al. 2014) |

**Supplementary Table S3** Summary of IC_50_, Cmax of compounds tested. All compounds were tested up to 20-fold of its Cmax unless otherwise stated. The IC_50_ values were calculated based on data shown in Figure 4.

|  | CYP1A2 | | CYP3A4 | |
| --- | --- | --- | --- | --- |
|  | - | IL-6 | - | IL-6 |
| TVX | 2.59±0.27 | 0.90±0.57 | 2.86±2.58 | 1.38±0.61 |
|  | 2.16±1.03 | 0.80±0.03 | 11.02±1.98 | 7.85±0.80 |
| APAP | 9.73±3.43 | 1.85±1.63 | 9.73±3.43 | 9.97±1.72 |
|  | 119.51±53.81 | 14.12±5.44 | 2.08±2.30 | 1.05±1.62 |

**Supplementary Table S4 Transcriptional level of CYP1A2 and CYP3A4 in HIVIL treated with drugs as well as exogenous IL-6. Exogenous IL-6 has been supplemented to the level in the LPS-treated vehicle control. Data presented as mean±S.D. of fold change relative to DMSO.**

|  | KEGG Pathway | padj | Size | Overlap | Leading Edge subset |
| --- | --- | --- | --- | --- | --- |
| 1 | Cytokine-cytokine receptor interaction | 5.50E-12 | 181 | 51 | TNFSF13, HGF, CCL2, IL21R, CCL5, CCL13, IL17RB, CCL8, FLT1 |
| 2 | NOD-like receptor signaling pathway | 5.99E-09 | 60 | 24 | CXCL1, TNF, CCL2, CCL5, CCL13, CCL8, MEFV, MAPK10, MAPK11 |
| 3 | Hematopoietic cell lineage | 1.22E-07 | 59 | 22 | GP1BA, CD33, TNF, ITGAM, ITGA4, KITLG, IL7R, MME, KIT |
| 4 | Toll-like receptor signaling pathway | 8.55E-06 | 85 | 24 | CD80, MYD88, CCL5, CD14, AKT3, NFKB1, PIK3R5, TLR5, CXCL11 |
| 5 | ECM-receptor interaction | 2.38E-05 | 79 | 22 | GP1BA, COL6A2, COL6A3, COL5A2, COL6A1, LAMA1, COMP, VTN, ITGA4 |
| 6 | Focal adhesion | 5.04E-05 | 193 | 38 | HGF, COMP, ITGA11, PGF, PARVG, FLT1, RELN, LAMC2, PIK3R5 |
| 7 | Intestinal immune network for IgA production | 6.81E-05 | 34 | 13 | CD80, TNFSF13, TNFSF13B, IL15RA, IL15, ITGA4, HLA-DPA1, HLA-DQA1, HLA-DQB1 |
| 8 | Complement and coagulation cascades | 0.0005 | 64 | 17 | F2, FGB, SERPIND1, SERPINA1, F10, CR1, F13A1, C7, FGG |
| 9 | Renin-angiotensin system | 0.0007 | 13 | 7 | ACE2, REN, AGT, ACE, ENPEP, CPA3 |
| 10 | Chemokine signaling pathway | 0.0011 | 162 | 30 | CCL2, CCL5, CCL13, CCL8, CCL17, AKT3, NFKB1, PIK3R5, SHC3 |
| 11 | Fc epsilon RI signaling pathway | 0.0024 | 67 | 16 | PRKCB, TNF, INPP5D, MAPK10, RAC2, MAPK11, MAP2K3, AKT3, GRB2 |
| 12 | Cytosolic DNA-sensing pathway | 0.0063 | 40 | 11 | CCL5, CASP1, NFKB1, IL18, POLR3D, RIPK3, PYCARD, IL1B, CCL4L2, IL6 |
| 13 | Cell adhesion molecules | 0.0068 | 118 | 22 | CD80, NLGN1, CD274, PDCD1LG2, ITGAM, ITGA4, SIGLEC1, ITGB7, SPN |
| 14 | JAK-STAT signaling pathway | 0.0179 | 104 | 19 | IL21R, AKT3, STAT5A, PIK3R5, CSF2RB, CSF3R, PTPN6, IL7R, IL12RB1 |
| 15 | Neuroactive ligand-receptor interaction | 0.0191 | 187 | 29 | PTGER4, GPR35, PTH2R, DRD4, NMUR1, PLG, C3AR1, GABRB2, GABRB1 |
| 16 | Arachidonic acid metabolism | 0.0274 | 42 | 10 | EPHX2, ALOX5, CYP2J2, AKR1C3, ALOX15, CBR3, GPX1, HPGDS, PLA2G2D |
| 17 | Calcium signaling pathway | 0.0294 | 151 | 24 | BST1, CALM1, ATP2A3, SPHK1, P2RX7, PDE1B, PDGFRA, PLCG2, PRKCB |
| 18 | B cell receptor signaling pathway | 0.0611 | 70 | 13 | PRKCB, PTPN6, INPP5D, RAC2, AKT3, GRB2, RASGRP3, NFKB1, PIK3R5 |
| 19 | Regulation of actin cytoskeleton | 0.0957 | 196 | 27 | ITGA11, ARPC4, CD14, ARPC5, PFN1, PIK3R5, CSK, WAS, FGFR2 |

**Supplementary Table S5** KEGG pathway overrepresentation analysis with “HIVIL-only” genes which were differentially expressed in HIVIL (cVeh+ vs. mVeh+) but unchanged in Padberg *et al.* (Padberg et al. 2020). We compared the co-culture induced changes in HIVIL (cVeh+ vs. mVeh+) against Padberg *et al.*’s indirect co-culture of HepG2 with THP-1. Regardless of differences in cell sources (IMR90 iHep vs. HepG2), sequencing platform (RNA-seq vs. microarray), and other differences between the studies, 1110 genes were identified in RankProd to be changed in the same direction by co-culturing in both studies. ‘HIVIL-only’ genes were identified by looking for differentially expressed genes in cVeh+ vs. mVeh+, then excluding genes differentially expressed in Padberg *et al.*, genes not expressed in both datasets and consistently regulated genes identified from RankProd. Of 15,797 genes in common between both datasets, 1393 fulfilled these criteria. Over-representation analysis was used to find KEGG pathways that HIVIL-only genes were involved in, against the background universe of genes expressed in both studies. Disease-specific pathways were excluded. Size refers to the number of genes in the pathway and expressed in the background. Overlap refers to the number of HIVIL-only genes in the leading edge driving the pathway enrichment. Leading edge highlights the top 9 genes driving the pathway enrichment.

|  | KEGG Pathway | padj | Size | Overlap | Leading Edge subset |
| --- | --- | --- | --- | --- | --- |
| 1 | Spliceosome | 5.69E-06 | 126 | 61 | LSM5, SF3B2, PRPF19, HNRNPA3, LSM6, USP39, NCBP1, MAGOH, SF3B3 |
| 2 | Arginine and proline metabolism | 0.001332 | 47 | 26 | SRM, AZIN2, GLS2, GLUD1, GLS, ASL, SMS, P4HA1, AOC1 |
| 3 | Autophagy - animal | 0.055656 | 20 | 12 | ATG4A, BECN1, ATG12, ATG4B, PRKAA2, PRKAA1, GABARAPL2, PIK3C3, ULK1 |
| 4 | RNA degradation | 0.057793 | 56 | 25 | LSM5, EXOSC2, LSM6, EXOSC3, EDC4, CNOT2, XRN1, ZCCHC7, ENO1 |
| 5 | Glutathione metabolism | 0.057793 | 45 | 21 | SRM, GGT1, SMS, PGD, MGST2, LAP3, GSTM1, GPX7, GSTA4 |

**Supplementary Table S6** KEGG pathway overrepresentation analysis with “HepG2+THP1-only” genes. ‘HepG2+THP1-only’ genes were identified by looking for differentially expressed genes in cocultured HepG2+THP-1 vs. monocultured HepG2, then excluding genes differentially expressed in HIVIL, genes not expressed in both datasets and consistently regulated genes identified from RankProd. Of 15,797 genes in common between both datasets, 4055 fulfilled these criteria.

|  | CYP1A2 | | CYP3A4 | |
| --- | --- | --- | --- | --- |
|  | - | IL-6 | - | IL-6 |
| TVX | 3.76±0.74 | 4.63±1.51 | 14.19±3.18 | 6.18±1.13 |
|  | 0.19±0.13 | 1.09±0.55 | 11.05±11.04 | 1.47±1.95 |
| APAP | 3.49±2.34 | 1.46±1.44 |  |  |
|  | 29.02±8.90 | 25.90±4.97 |  |  |

**Supplementary Table S7** Transcriptional level of CYP1A2 and CYP3A4 in iPSC (F4)-derived HIVIL treated with drugs and exogenous IL-6. **Exogenous IL-6 was supplemented to the level in the LPS-treated vehicle control. Data presented as mean±S.D. of fold change relative to DMSO.**

| Target Gene | Gene Name | Accession |
| --- | --- | --- |
| GAPDH | glyceraldehyde-3-phosphate dehydrogenase | NM_001256799.2 |
| AFP | Alpha fetoprotein | NM_000295 |
| AAT | Alpha-1 antitrypsin | NM_001134 |
| ASGPR | Asialoglycoprotein Receptor | NM_001671 |
| ALB | albumin | NM_000477.3 |
| CYP1A2 | Cytochrome P450 1A2 | NM_000761.5 |
| CYP3A4 | Cytochrome P450 3A4 | NM_017460.5 |
| CYP2B6 | Cytochrome P450 2B6 | NM_000767.4 |
| CYP2C9 | Cytochrome P450 2C9 | NM_000771.3 |
| UGT1A3 | UDP-glucuroosyltransferase 1A3 | NM_019093.2 |
| GST1A2 | Glutathione S-Transferase | NM_000846.3 |
| MRP2 | Multidrug resistance-associated protein 2 | NM_000392 |
| CD14 | Cluster of differentiation 14 | NM_001174105.1 |
| CD32 | Cluster of differentiation 32 | NM_001136219.1 |
| CD68 | Cluster of differentiation 68 | NM_001251.2 |
| CD163 | Cluster of differentiation 163 | NM_203416.3 |
| ID1 | Inhibitor of DNA-binding protein 1 | NM_002165.3 |
| ID3 | Inhibitor of DNA-binding protein 3 | NM_002167.4 |
| CLEC1B | C-type lectin domain family 1 member B | NM_016509.3 |
| VSIG4 | V-set and immunoglobulin domain containing 4 | NM_007268.2 |
| TIMD4 | T cell immunoglobulin and mucin domain containing | NM_001146726.1 |

**Supplementary Table S8 Accession number of genes tested with qPCR.**

# Supplementary Methods

*Modifications to iKC differentiation protocol:* Briefly, in the original protocol, pre-macrophages (pre-M) produced from embryonic bodies under M-SCF and IL-3 treatment were subjected to 7-day exposure of medium containing 5% FBS and hepatic cues from hepatocyte conditioned medium. Media was changed every three days during the seven days pre-M to iKCs differentiation period. Serum-free media was used for media change. In the re-optimized protocol, pre-M generation stayed unchanged; however, both M-SCF and serum were used on the first day of differentiation. Serum was removed from the differentiation media during media change after three days. Growth factors used in both protocols, except macrophage colony stimulation factor (M-CSF, STEMCELL Technologies), were purchased from R&D Systems (Minneapolis, MN, USA).

*Validation of serum and M-CSF addition:* According to the original protocol (Tasnim et al. 2019), a mixture of primary hepatocyte conditioned medium (PHCM) and FBS-containing Advanced DMEM (plus supplements) was used for iKC differentiation (termed as S/-). FBS-free Advanced DMEM (plus supplements) alone (termed as -/-), and M-CSF-added medium (termed as S/M, 50 ng/mL) were tested to validate the effects of serum and M-CSF on iKCs differentiation. The confluency changes were captured every 2 days, and processed using MATLAB^®^. Phase contrast images were imported into the MATLAB Image Processing Toolbox (Mathworks) for further processing. The raw images were first processed through a rolling ball background subtraction to even out light intensity. Then, the processed images were transformed into binary images using adaptive thresholding. To fully trace each cell and eliminate any background noise, morphological opening and dilation were performed to the binary mask. Each object with 8 pixels connection in the binary image was recognized as cell region. Thereafter, the confluency was defined as the total number of pixels in cell region divided by the total pixels of the corresponding image.

*THP-1 maintenance and differentiation:* THP-1 monocytic lineage was maintained in RPMI 1640 with 10% FBS. For differentiation to macrophages, suspension cells were collected and subjected to 2-day phorbol 12-myristate 13-acetate (PMA) treatment (50 ng/ml), followed by a 1-day PMA-free medium resting. THP-1 macrophages were trypsinzed and used for co-culture as described in *Material and methods*.

*Cell viability assays:* Cell viability was examined with AlamarBlue^TM^ cell viability assay (Thermo Fisher Scientific Inc.) according to manufacturer’s instructions. Briefly, cells were exposed to AlamarBlue^TM^ working solution, which was diluted 10-fold using PBS containing 2 mg/mL of glucose, for 1 hour. Color change in the solution was measured using fluorescence (Ex: 530nm, Em: 590nm) in Tecan Microplate Reader M1000 PRO. The viability of dosed cells was presented as percentage of the corresponding vehicle control.

*Enzyme-linked immunosorbent assay (ELISA) for measurement of cytokines:* Interleukin-6 (IL-6), interleukin-10 (IL-10) and tumor necrosis factor alpha (TNFα) levels in the media were measured using the corresponding human-based ELISA kits (Abcam, Cambridge, United Kingdom) according to manufacturer’s instructions. The cytokine production from dosed cells was normalized with the viability value from the same well. The normalized cytokine level upon drug treatments was further presented as percentage of DMSO+LPS. Normalization to viability per well ensures that any changes in cytokines are not due to changes in cell numbers upon drug treatment and normalization to DMSO+LPS allows better comparison between different batches of experiments. This normalization approach has been used in previous studies (Rose et al. 2016).

*Quantitative real time PCR (qPCR):* Total RNA was extracted from cells using RNeasy Plus Micro-kit (Qiagen, Hilden, Germany), followed by quantification using a NanoDrop^TM^ ND-1000 Spectrophotometer and conversion to cDNA using iScript cDNA synthesis kit (Bio-Rad Laboratories, Hercules, USA). qPCR was performed in 7000 Fast Real-Time PCR System (Applied Biosystems, Foster City, USA) with FastStart Universal SYBR Green Master (Rox) (Roche, Basel, Switzerland) and primers from GeneCopoeia, Inc. (Rockville, MD, USA). Gyceraldehyde-3-phosphate dehydrogenase (GAPDH) served as internal control. Accession numbers of tested genes are listed in Supplementary Table S8.

*Albumin and urea measurement:* Supernatant from HIVIL cultured on Day 1 and Day 5 was collected for albumin and urea assays. Albumin secretion was measured using a human albumin ELISA quantitation kit quantification kit (Bethyl Laboratories, Inc., Montgomery, TX, USA) as per manufacturer's protocol. Urea production was measured using Direct Urea Nitrogen Color Reagent and Direct Urea Nitrogen Acid Reagent (Standbio Laboratory, Boerne, TX, USA) as described in (Tasnim et al. 2015). All functional data was normalized to the number of cells seeded that was quantified using the Quant-iT PicoGreen dsDNA Assay Kit (Invitrogen, Singapore).

*CYP activity measurement:* P450-Glo^TM^ Assays (Promega, Madison, WI, USA) were used to measure CYP1A2, CYP3A4 and CYP2B6 in HIVIL according to the manufacturer’s instruction.

*RNA seq and analysis:* After 48 hr of drug treatment, iHeps cultured in mono-culture and HIVIL were both subjected to TrypLE^TM^ dissociation, and subsequently sorted with magnetic separation with the CD163 Microbead kit (Miltenyi Biotec, Bergisch Gladbach, Germany) to obtain only iHeps. Flow-through was collected, pelleted down and snap-frozen in liquid nitrogen, before storage at -80°C. Lysates were sent to the Genome Research Informatics and Data Science platform (Agency for Science, Technology and Research, Singapore). RNA extraction was done using TRIzol and analyzed on a Tapestation 4200 to determine RNA Integrity Number (RIN). All sequenced samples included for further analysis had RIN > 8. Illumina Stranded mRNA multiplexing was done with unique 96 dual indices, and all samples sequenced on the same lane of NovaSeq 6000 S4 flowcell. Sequenced reads were trimmed for adaptor sequence using Trim Galore v0.6.4, then aligned to human genome build GRCh38 using STAR_2.6.1d. Aligned reads are summarized into count matrices using featureCounts v1.6.4 with the gene annotation file from GENCODE v37.

Data normalization was performed using DESeq2 v1.32.0 (Love et al. 2014) on the R programming language v4.1.0 (Core Team 2021; Team 2021). Gene enrichment analysis was done with fgsea v1.18 (Korotkevich et al. 2021) with gene sets downloaded from MSigDB and KEGG pathway visualization done via pathview v1.32 (Kanehisa et al. 2021; Luo and Brouwer 2013). Other plots were produced using ggplot2 v3.3.5 and scatterplot3d v0.3-41 (Ligges and Maechler 2003). The RNA-Seq dataset has been deposited in NCBI under accession number GSE189320.

Data from another co-culture study was downloaded from GEO accession GSE140141 (Padberg et al. 2020). Genes in common between the two datasets were identified by gene symbols. RankProd v3.20.0 (Carratore et al. 2017) was used to identify genes changed in the same direction by co-culture in both studies via a non-parametric rank-based method. ‘HIVIL-only’ genes were identified by looking for differentially expressed genes in cVeh+ vs. mVeh+, then excluding genes differentially expressed in GSE140141, genes not expressed in both datasets and consistently regulated genes identified from RankProd. Simple over-representation analysis was used to find KEGG pathways that HIVIL-only genes were involved in, against the background universe of genes expressed in both studies.

The datasets used in the dimension reduction plot were downloaded from GSE140141 (Padberg et al. 2020), GSE105019 (Fu et al. 2019) and GSE112330 (Xie et al. 2019). All datasets were joined together by common gene symbol identifiers. Multiple probes that map to the same gene symbol were combined by taking the median of probe reads. The joined dataset was log-normalized (divided by the total count, multiplied by 1 million and natural-log transformed using log1p) and center-scaled. From the scaled data, the 3000 most variable genes across datasets were selected for dimensionality reduction by Uniform Manifold Approximation and Projection (UMAP) algorithm using the R packages umap v0.2.7 (Konopka 2020) and Seurat v4.1.0 (Hao et al. 2021).

# Bibliography

Ademisoye AA, Soyinka JO, Olawoye SO, et al. (2018) Induction of Amodiaquine Metabolism by Rifampicin Following Concurrent Administration in Healthy Volunteers. J Explor Res Pharmacol 3(3):71-77

Agabeyoglu I, Incecayir T (2009) Pharmacokinetic Modelling of Lamotrigine from Plasma Concentrations in Healthy Volunteers. J Bioanal Biomed 1:041-045

Bareggi S, Cerutti R, Pirola R, Riva R, Cisternino M (1987) Clinical pharmacokinetics and metabolism of pyrazinamide in healthy volunteers. Arzneimittel-Forschung 37(7):849

Bekker Z, Walubo A, du Plessis JB (2012) The role of the immune system in nevirapine-induced subclinical liver injury of a rat model. Int Scholarly Res Notices 2012

Bekker Z, Walubo A, Du Plessis JB (2016) Changes in IL-2 and IL-10 during chronic administration of isoniazid, nevirapine, and paracetamol in rats. Adv Pharmacol Sci 2016

Bonzo JA, Rose K, Freeman K, et al. (2015) Differential effects of trovafloxacin on TNF-α and IL-6 profiles in a rat hepatocyte–Kupffer cell coculture system. Appl In Vitro Toxicol 1(1):45-54

Cammett AM, MacGregor TR, Wruck JM, et al. (2009) Pharmacokinetic assessment of nevirapine and metabolites in human immunodeficiency virus type 1-infected patients with hepatic fibrosis. Antimicrob Agents Chemother 53(10):4147-4152

Carratore FD, Jankevics A, Eisinga R, Heskes TM, Hong F, Breitling R (2017) RankProd 2.0: a refactored bioconductor package for detecting differentially expressed features in molecular profiling datasets. Bioinformatics 33:2774 - 2775

Chen M, Suzuki A, Thakkar S, Yu K, Hu C, Tong W (2016) DILIrank: the largest reference drug list ranked by the risk for developing drug-induced liver injury in humans. Drug Discov Today 21(4):648-653

Core Team R (2021) R: A language and environment for statistical computing. R Foundation for Statistical Computing, Vienna, Austria

Dik B, Sonmez G, Faki HE, Bahcivan E (2018) Sulfasalazine treatment can cause a positive effect on LPS-induced endotoxic rats. Exp Animals 67(4):403-412

Fu G-B, Huang W-J, Zeng M, et al. (2019) Expansion and differentiation of human hepatocyte-derived liver progenitor-like cells and their use for the study of hepatotropic pathogens. Cell Res 29(1):8-22 doi:10.1038/s41422-018-0103-x

Gandhi A, Guo T, Shah P, Moorthy B, Ghose R (2013) Chlorpromazine-induced hepatotoxicity during inflammation is mediated by TIRAP-dependent signaling pathway in mice. Toxicol Appl Pharmacol 266(3):430-438

Ganey PE, Luyendyk JP, Maddox JF, Roth RA (2004) Adverse hepatic drug reactions: inflammatory episodes as consequence and contributor. Chem-Biol Interact 150(1):35-51

Giustarini G, Vrisekoop N, Kruijssen L, et al. (2019) Trovafloxacin-induced liver injury: lack in regulation of inflammation by inhibition of nucleotide release and neutrophil movement. Toxicol Sci 167(2):385-396

Guo S, Al-Sadi R, Said HM, Ma TY (2013) Lipopolysaccharide causes an increase in intestinal tight junction permeability in vitro and in vivo by inducing enterocyte membrane expression and localization of TLR-4 and CD14. Am J Pathol 182(2):375-387

Hao Y, Hao S, Andersen-Nissen E, et al. (2021) Integrated analysis of multimodal single-cell data. Cell 184(13):3573-3587. e29

Hassan HM, Guo H, Yousef BA, et al. (2016) Role of inflammatory and oxidative stress, cytochrome P450 2E1, and bile acid disturbance in rat liver injury induced by isoniazid and lipopolysaccharide cotreatment. Antimicrobial agents and chemotherapy 60(9):5285-5293

Heidari R, Ahmadi F, Rahimi HR, et al. (2019) Exacerbated liver injury of antithyroid drugs in endotoxin-treated mice. Drug Chem Toxicol 42(6):615-623

Higuchi S, Yano A, Takai S, et al. (2012) Metabolic activation and inflammation reactions involved in carbamazepine-induced liver injury. Toxicol Sci 130(1):4-16

Ho S, McLachlan A, Chen T, Hibbs D, Fois R (2015) Relationships Between Pharmacovigilance, Molecular, Structural, and Pathway Data: Revealing Mechanisms for Immune‐Mediated Drug‐Induced Liver Injury. CPT: Pharmacomet Syst Pharmacol 4(7):426-441

Honarmand H, Abdollahi M, Ahmadi A, et al. (2012) Randomized trial of the effect of intravenous paracetamol on inflammatory biomarkers and outcome in febrile critically ill adults. DARU J Pharm Sci 20(1):12

Hong-mei W, Xue-hua J, Lin S, Ling HYaW (2013) Studies on determination of sulfasalazine and sulfapyridine in human plasma by HPLC and pharmacokinetics in human volunteers [J]. Chinese Journal of Antibiotics 3

Jiang J, Messner S, Kelm JM, et al. (2019) Human 3D multicellular microtissues: An upgraded model for the in vitro mechanistic investigation of inflammation-associated drug toxicity. Toxicol Lett 312:34-44

Jiang X, Wang N, Zhang Z-J, Tian Y, Chen Y (2008) Pharmacokinetics and comparative bioavailability of two terbinafine hydrochloride formulations after single-dose administration in Chinese healthy subjects. Arzneimittelforschung 58(07):363-366

Kanehisa M, Furumichi M, Sato Y, Ishiguro-Watanabe M, Tanabe M (2021) KEGG: integrating viruses and cellular organisms. Nucleic Acids Res 49(D1):D545-d551 doi:10.1093/nar/gkaa970

Kang B-S, Chung E-Y, Yun Y-P, et al. (2001) Inhibitory effects of anti-inflammatory drugs on interleukin-6 bioactivity. Biol Pharm Bull 24(6):701-703

Kaplowitz N (2005) Idiosyncratic drug hepatotoxicity. Nat Rev Drug Discov 4(6):489-99 doi:10.1038/nrd1750

Konopka T (2020) UMAP: Uniform Manifold Approximation and Projection. R package version 0.2.7.0. edn

Korotkevich G, Sukhov V, Budin N, Shpak B, Artyomov MN, Sergushichev A (2021) Fast gene set enrichment analysis. BioRxiv:060012

Li F, Cao L, Parikh S, Zuo R (2020) Three-Dimensional Spheroids With Primary Human Liver Cells and Differential Roles of Kupffer Cells in Drug-Induced Liver Injury. J Pharm Sci 109(6):1912-1923 doi:10.1016/j.xphs.2020.02.021

Li J, Yao H-W, Jin Y, et al. (2002) Pharmacokinetics of leflunomide in Chinese healthy volunteers. Acta Pharmacol Sinica 23(6):551-555

Ligges U, Maechler M (2003) Scatterplot3d - an R package for visualizing multivariate data. Journal of Statistical Software 008:1-20

Love MI, Huber W, Anders S (2014) Moderated estimation of fold change and dispersion for RNA-seq data with DESeq2. Genome Biol 15(12):550 doi:10.1186/s13059-014-0550-8

Luo W, Brouwer C (2013) Pathview: an R/Bioconductor package for pathway-based data integration and visualization. Bioinformatics 29(14):1830-1831

Mahdy A, Galley HF, Abdel‐Wahed M, El‐Korny K, Sheta S, Webster NR (2002) Differential modulation of interleukin‐6 and interleukin‐10 by diclofenac in patients undergoing major surgery. British Journal of Anaesthesia 88(6):797-802

Manna SK, Aggarwal BB (1999) Immunosuppressive leflunomide metabolite (A77 1726) blocks TNF-dependent nuclear factor-κB activation and gene expression. J Immunol 162(4):2095-2102

Mendes GD, Bittencourt S, Vespasiano CFP, et al. (2014) Propylthiouracil quantification in human plasma by high-performance liquid chromatography coupled with electrospray tandem mass spectrometry: Application in a bioequivalence study. J Chromatogr B 969:19-28

Nguyen TV, Ukairo O, Khetani SR, et al. (2015) Establishment of a hepatocyte-kupffer cell coculture model for assessment of proinflammatory cytokine effects on metabolizing enzymes and drug transporters. Drug Metab Dispos 43(5):774-785

Padberg F, Hering H, Luch A, Zellmer S (2020) Indirect co-cultivation of HepG2 with differentiated THP-1 cells induces AHR signalling and release of pro-inflammatory cytokines. Toxicol In Vitro 68:104957 doi:10.1016/j.tiv.2020.104957

Radi ZA, Koza-Taylor PH, Bell RR, et al. (2011) Increased serum enzyme levels associated with kupffer cell reduction with no signs of hepatic or skeletal muscle injury. Am J Pathol 179(1):240-247

Rafal P. Witek KBA, Manda A. Edwards, Adam M. Farmer, Kimberly M. Freeman, Erica Deibert, Tania Bembridge, Jonathan P. Jackson,, Cornelia Smith JS, Stephen S. Ferguson, Mark J. Powers (2010) Co-culture of Hepatocytes and Kupffer Cells as a Model for Liver Inflammation.

Reunanen M, Heinonen E, Nyman L, Anttila M (1992) Comparative bioavailability of carbamazepine from two slow-release preparations. Epilepsy Res 11(1):61-66

Rojanasthien N, Chaichana N, Teekachunhatean S, Kumsorn B, Sangdee C, Chankrachang S (2007) Effect of doses on the bioavailability of phenytoin from a prompt-release and an extended-release preparation: single dose study. Journal Medical Association of Thailand 90(9):1883

Rose KA, Holman NS, Green AM, Andersen ME, LeCluyse EL (2016) Co-culture of Hepatocytes and Kupffer Cells as an In Vitro Model of Inflammation and Drug-Induced Hepatotoxicity. J Pharm Sci 105(2):950-964 doi:10.1016/s0022-3549(15)00192-6

Shaw PJ, Ganey PE, Roth RA (2010) Idiosyncratic drug-induced liver injury and the role of inflammatory stress with an emphasis on an animal model of trovafloxacin hepatotoxicity. Toxicol Sci 118(1):7-18

Steuerwald NM, Foureau DM, Norton HJ, et al. (2013) Profiles of serum cytokines in acute drug-induced liver injury and their prognostic significance. Plos One 8(12)

Tasnim F, Phan D, Toh YC, Yu H (2015) Cost-effective differentiation of hepatocyte-like cells from human pluripotent stem cells using small molecules. Biomaterials 70:115-25 doi:10.1016/j.biomaterials.2015.08.002

Tasnim F, Xing J, Huang X, et al. (2019) Generation of mature kupffer cells from human induced pluripotent stem cells. Biomaterials 192:377-391

Team R (2021) RStudio: Integrated Development Environment for R. RStudio, PBC, Boston, MA

Thakkar S, Li T, Liu Z, Wu L, Roberts R, Tong W (2020) Drug-induced liver injury severity and toxicity (DILIst): Binary classification of 1279 drugs by human hepatotoxicity. Drug Discov Today 25(1):201-208

Verma S, Kaplowitz N (2009) Diagnosis, management and prevention of drug-induced liver injury. Gut 58(11):1555-1564

Wahl C, Liptay S, Adler G, Schmid RM (1998) Sulfasalazine: a potent and specific inhibitor of nuclear factor kappa B. J Clini Invest 101(5):1163-1174

Xie B, Sun D, Du Y, et al. (2019) A two-step lineage reprogramming strategy to generate functionally competent human hepatocytes from fibroblasts. Cell Res 29(9):696-710 doi:10.1038/s41422-019-0196-x

Xu JJ, Henstock PV, Dunn MC, Smith AR, Chabot JR, de Graaf D (2008) Cellular imaging predictions of clinical drug-induced liver injury. Toxicol Sci 105(1):97-105 doi:10.1093/toxsci/kfn109

Yamamoto T, Kaizu C, Kawasaki T, et al. (2008) Macrophage colony-stimulating factor is indispensable for repopulation and differentiation of Kupffer cells but not for splenic red pulp macrophages in osteopetrotic (op/op) mice after macrophage depletion. Cell Tissue Res 332(2):245-256

Yao H-W, Li J, Chen J-Q, Xu S-Y (2004) Leflunomide attenuates hepatocyte injury by inhibiting Kupffer cells. World J Gastroenterol 10(11):1608

Zou W, Beggs KM, Sparkenbaugh EM, et al. (2009) Sulindac metabolism and synergy with tumor necrosis factor-α in a drug-inflammation interaction model of idiosyncratic liver injury. The Journal of pharmacology and experimental therapeutics 331(1):114-121
